# On Robust Association Testing for Quantitative Traits and Rare Variants

**DOI:** 10.1534/g3.116.035485

**Published:** 2016-09-27

**Authors:** Peng Wei, Ying Cao, Yiwei Zhang, Zhiyuan Xu, Il-Youp Kwak, Eric Boerwinkle, Wei Pan

**Affiliations:** * Department of Biostatistics, The University of Texas MD Anderson Cancer Center, Houston, Texas 77030; † Human Genetics Center, The University of Texas School of Public Health, Houston, Texas 77030; ‡ Division of Biostatistics, School of Public Health, University of Minnesota, Minneapolis, Minnesota 55455; § Human Genome Sequencing Center, Baylor College of Medicine, Houston, Texas 77030

**Keywords:** SKAT, associate testing, next-generation sequencing, rare variants, robustness

## Abstract

With the advance of sequencing technologies, it has become a routine practice to test for association between a quantitative trait and a set of rare variants (RVs). While a number of RV association tests have been proposed, there is a dearth of studies on the robustness of RV association testing for nonnormal distributed traits, *e.g.*, due to skewness, which is ubiquitous in cohort studies. By extensive simulations, we demonstrate that commonly used RV tests, including sequence kernel association test (SKAT) and optimal unified SKAT (SKAT-O), are not robust to heavy-tailed or right-skewed trait distributions with inflated type I error rates; in contrast, the adaptive sum of powered score (aSPU) test is much more robust. Here we further propose a robust version of the aSPU test, called aSPUr. We conduct extensive simulations to evaluate the power of the tests, finding that for a larger number of RVs, aSPU is often more powerful than SKAT and SKAT-O, owing to its high data-adaptivity. We also compare different tests by conducting association analysis of triglyceride levels using the NHLBI ESP whole-exome sequencing data. The QQ plots for SKAT and SKAT-O were severely inflated (*λ* = 1.89 and 1.78, respectively), while those for aSPU and aSPUr behaved normally. Due to its relatively high robustness to outliers and high power of the aSPU test, we recommend its use complementary to SKAT and SKAT-O. If there is evidence of inflated type I error rate from the aSPU test, we would recommend the use of the more robust, but less powerful, aSPUr test.

Thanks to the rapidly decreasing cost of the next-generation sequencing (NGS) technology, whole-exome sequencing (WES) and whole-genome sequencing (WGS) have been performed in many deeply phenotyped prospective cohort studies and electronic health record (EHR)-based cohorts of tens of thousands of individuals. Completed and ongoing large-scale WES and WGS sequencing efforts include the National Heart, Lung, and Blood Institute (NHLBI) Exome Sequencing Project (ESP) (Crosby *et al.* 2014), Trans-Omics for Precision Medicine (TOPMed) Program (Abecasis *et al.* 2015), the NHGRI Genome Sequencing Program (GSP), the UK10K project (UK10K Consortium 2015), and the Geisinger MyCode project (Mukherjee *et al.* 2015), to name a few. This big wave of sequencing data provides researchers with unprecedented opportunities to investigate low frequency [minor allele frequency (MAF) between 1 and 5%] and rare (MAF <1%) single nucleotide variants (SNVs) in association with complex phenotypes and diseases (Yi *et al.* 2011; Schaid *et al.* 2013; Lee *et al.* 2014). An example of the initial successes is the discovery of rare functional variants in *APOC3* associated with lower plasma triglyceride levels and a reduced risk of coronary heart disease (Crosby *et al.* 2014).

Many phenotypes such as triglyceride and fasting glucose collected in population-based cohort studies are quantitative and may not follow a normal distribution, as explicitly or implicitly assumed in most existing statistical methods for rare variant (RV)-based association testing (Bansal *et al.* 2010; Fan *et al.* 2015). However, there is a dearth of literature on the robustness of RV tests to the nonnormality of the observed traits, *e.g.*, due to skewness, which is expected to be ubiquitous in cohort studies. In particular, we find that commonly used RV tests, including the sequence kernel association test (SKAT) (Wu *et al.* 2011) and SKAT-O test (Lee *et al.* 2012), are very sensitive to quantitative trait’s deviation from normality and can have severely inflated association p-values. For example, when applied to the ESP WES data in association with plasma triglyceride levels, as described in detail later on, SKAT and SKAT-O had globally inflated quantile–quantile (QQ) plots with genomic control (GC; Devlin and Roeder 1999) λ=1.89 and 1.78, respectively. In addition to the case study of RV-triglyceride association testing, here we have conducted extensive simulation studies to investigate the performance of several commonly used RV tests, including the burden test (Li and Leal 2008), SKAT, and SKAT-O, as well as our recently proposed adaptive sum of powered score (aSPU) test (Pan *et al.* 2014), in the presence of nonnormal quantitative traits. We have also studied and compared some commonly used *ad hoc* strategies to deal with nonnormal traits, such as natural logarithm transformation, inverse normal transformation, Winsorizing, trimming, and minor allele count (MAC) thresholding. Although we find that the aSPU test is more robust than SKAT and SKAT-O, it can sometimes suffer from inflated type I error rates in the presence of a few contaminated observations. In response, we further propose a robust version of the aSPU test, called aSPUr. While the traditional variant-by-variant association test for common SNVs (MAF >5%) has been shown to be robust to nonnormal distributed traits (Cao *et al.* 2014), here we demonstrate that RV association testing can be very sensitive to quantitative trait’s subtle deviation from normality. Based on type I error control and statistical power considerations, we further provide practitioners with some general guidelines and a new robust test to deal with nonnormal quantitative traits.

## Methods

### Review of existing RV tests

We first review our recently proposed class of sum of powered score (SPU) tests and their adaptive version called aSPU test (Pan *et al.* 2014). The former include the burden and SKAT tests as special cases. We then introduce a new robust version of the SPU and aSPU tests, denoted as SPUr and aSPUr. Consider a linear model for a quantitative trait,

$$Y_{i}=\beta_{0}+\sum_{j=1}^{k}X_{ij}\beta_{j}+\epsilon_{i},$$

where $Y_{i}$ is the trait for subject *i*, $X_{ij}$ is the MAC (coded as 0, 1, or 2) of SNV *j* for subject *I*, and the error term $\epsilon_{i}$ is assumed to have a distribution with mean 0 and a constant variance $\sigma^{2}.$ The main interest is to test $H_{0}:$ $\beta ={\left(\beta_{1},\ldots ,\beta_{k}\right)}^{\prime }=0,$ *i.e.*, none of the *k* variants in a set is associated with the phenotype. The score vector is

$$U=\sum_{i=1}^{n}\left(Y_{i}-\bar{Y}\right)X_{i},$$

and its covariance matrix is $V=Cov\left(U\right)=\sigma^{2}\sum_{i=1}^{n}\left(X_{i}-\bar{X}\right){\left(X_{i}-\bar{X}\right)}^{\prime },$ which can be consistently estimated by $\hat{V}=\sum_{i=1}^{n}{\left(Y_{i}-\bar{Y}\right)}^{2}\left(X_{i}-\bar{X}\right){\left(X_{i}-\bar{X}\right)}^{\prime }.$ In fact, for any generalized linear models (GLMs) with a canonical link function, the score vector *U* remains the same as the above.

Pan *et al.* (2014) proposed a class of SPU tests, for an integer γ≥1,

$$T_{\mathrm{SPU}}=T_{\mathrm{SPU}\left(\gamma \right)}\left(U\right)=\sum_{j=1}^{k}U_{j}^{\gamma }.$$

Note that when γ=1 and γ=2, the SPU test is equivalent to the burden test and the SKAT test under the linear kernel with equal RV weighting, respectively. Importantly, as *γ* increases, the SPU(*γ*) test puts more weights on the larger components of *U* while gradually ignoring the remaining components. In particular, we have

$$T_{\mathrm{SPU}\left(\gamma \right)}\propto {\left\|U\right\|}_{\gamma }={\left(\sum_{j=1}^{k}{\left|U_{j}\right|}^{\gamma }\right)}^{1/\gamma }\to {\left\|U\right\|}_{\infty }=\overset{k}{\underset{j=1}{\max}}\left|U_{j}\right|,\;\text{as}\;\gamma \to \infty .$$

As will be shown, since the SPU tests are based on resampling methods to calculate their p-values, they are invariant to monotone transformations, such as ${\left(.\right)}^{1/\gamma }.$ That is, we can define $T_{\mathrm{SPU}\left(\infty \right)}=\max_{j=1}^{k}\left|U_{j}\right|,$ which uses only the largest component of |U| and does not aggregate information from other RVs. More generally, as we increase the value of *γ*, we put higher and higher weights on the larger components of *U*, effectively realizing RV selection. On the other hand, an even integer of *γ* automatically eliminates the effects of different signs of $U_{j}$’s, avoiding power loss of the burden test in the presence of different association directions. However, an odd integer of *γ* might be more suitable, as in the SPU(1) or burden test, when the associations are all in the same direction.

Without covariates, Pan *et al.* (2014) proposed using permutations to obtain p-values for the SPU tests. With covariates, the parametric bootstrap (or, alternatively, permuting residuals) can be performed. Briefly, we fit a null model under $H_{0}$ and obtain the residuals, then we randomly permute the residuals and add them to the estimated means of the traits from the null model, obtaining a new set of null traits $Y^{\left(b\right)}.$ We use the null traits $Y^{\left(b\right)}$ to obtain a null statistic $T_{\mathrm{SPU}}^{\left(b\right)}=T_{\mathrm{SPU}}\left(Y^{\left(b\right)}\right).$ We repeat the above process *B* times, and calculate the p-value as $[\sum_{b=1}^{B}I(\left|T_{\mathrm{SPU}}^{\left(b\right)}\right|\geq \left|T_{\mathrm{SPU}}|\right)+1]/\left(B+1\right).$

Since the power of an SPU(*γ*) test depends on the choice of *γ* while the optimal choice of *γ* depends on the unknown true association pattern of the RVs to be tested, it would be desirable to data-adaptively choose the value of *γ*. For this purpose, Pan *et al.* (2014) proposed an adaptive SPU (aSPU) test to combine information across multiple SPU tests with various values of *γ*. Suppose that we have some candidate values of *γ* in Γ, *e.g.*, Γ={1,2,3,…,8,∞} as used in our later simulation experiments, and suppose that the p-value of the SPU(γ) test is $P_{\mathrm{SPU}\left(\gamma \right)},$ then our combining procedure is to take the minimum p-value:

$$T_{\mathrm{aSPU}}=\underset{\gamma \in \Gamma }{\min}P_{\mathrm{SPU}\left(\gamma \right)}.$$

Of course, $T_{\mathrm{aSPU}}$ is no longer a genuine p-value; as for the SPU tests, we recourse to a resampling method to estimate its p-value. As before, first we simulate *B* independent copies of the null traits $Y^{\left(b\right)}$ by the parametric bootstrap for b=1,2,…,B. We then calculate the corresponding SPU test statistics $T_{\mathrm{SPU}\left(\gamma \right)}^{\left(b\right)}$ and their p-values $p_{\gamma }^{\left(b\right)}=\left[\sum_{b_{1}\neq b}I\left(T_{\mathrm{SPU}\left(\gamma \right)}^{\left(b_{1}\right)}\geq T_{\mathrm{SPU}\left(\gamma \right)}^{\left(b\right)}\right)+1\right]/B.$ Thus, we have $T_{\mathrm{aSPU}}^{\left(b\right)}=\min_{\gamma \in \text{Γ}}p_{\gamma }^{\left(b\right)},$ and the final p-value of the aSPU test is $P_{\mathrm{aSPU}}=\left[\sum_{b=1}^{B}I\left(T_{\mathrm{aSPU}}^{\left(b\right)}\leq T_{\mathrm{aSPU}}\right)+1\right]/\left(B+1\right).$ We used B=1000 in our simulation experiments. Note that, we can first use a smaller B=1000 or so to scan a genome, then use a larger *B* to test on a few genes or regions that pass the significance criterion (*e.g.*, p-value <5/B) in the first step.

### New tests: robust SPU and aSPU tests

A potential problem with the above Gaussian likelihood-based approach is its nonrobustness to outliers, which can be caused by non-Gaussian errors $\epsilon_{i}$ or contaminated traits $Y_{i}.$ Consider a situation where we observe a singleton for RV *j*; that is, say $X_{1j}=1$ and all other $X_{ij}=0$ for i>1. Then the *j*th component of the score vector is $U_{j}=Y_{1}-\bar{Y},$ which will be largely influenced by a single observation $Y_{1}.$ As to be shown later, in such a situation, if $Y_{1}$ is contaminated or measured with error, then we may have inflated type I errors. To overcome the problem, we propose using a robust regression method. Rather than using the Gaussian-based likelihood, we propose using the Huber loss with the corresponding score vector $U_{H}=\sum_{i=1}^{n}U_{H,i.}$ with $X_{i.}={\left(X_{i1},\ldots ,X_{ik}\right)}^{\prime }$ and

$$U_{H,i.}=\{\begin{array}{ll} X_{i.}\left(Y_{i}-\bar{Y}\right)/\hat{\sigma } & \text{if}\;\left|Y_{i}-\bar{Y}\right|/\hat{\sigma }\leq c,\\ cX_{i.}\text{sign}\left(Y_{i}-\bar{Y}\right) & \text{otherwise}, \end{array}$$

where c=1.345 is chosen to maintain a high efficiency for a normal error (*i.e.*, trait) distribution, and $\hat{\sigma }$ is an estimate of *σ* (Jureckova and Picek 2006). Under $H_{0}$ we can use the median absolute deviation (MAD) as a robust estimate of *σ*. $\hat{\sigma }={\text{Median}}_{1\leq i\leq n}\left|Y_{i}-{\text{Median}}_{1\leq i^{\prime }\leq n}Y_{i^{\prime }}\right|/0.6745.$ It is clear that the truncation of $\left|Y_{i}-\bar{Y}\right|/\hat{\sigma }$ at a constant *c* eliminates or alleviates the undue influence of outlying $Y_{i}$’s.

We define a robust SPU (SPUr) test for a given γ≥1 as

$$T_{\mathrm{SPUr}\left(\gamma \right)}=\sum_{j=1}^{k}U_{H,j}^{\gamma }.$$

With various values of γ≥1, we obtain a class of the SPUr tests. Accordingly we define an adaptive robust SPU (aSPUr) test as

$$T_{\mathrm{aSPUr}}=\underset{\gamma \in \Gamma }{\min}P_{\mathrm{SPUr}\left(\gamma \right)},$$

where $P_{\mathrm{SPUr}\left(\gamma \right)}$ is the p-value of the SPUr(*γ*) test, and we use Γ={1,2,…,8,∞} as before. The p-values of the SPUr and aSPUr tests are obtained in the same way as for the SPU and aSPU tests described earlier.

Alternatively, based on some initial estimate ${\hat{\beta }}^{0}$ (*e.g.*, the least squares or least absolute deviation estimate) of *β*, we define residuals $e_{i}\left({\hat{\beta }}^{0}\right)=Y_{i}-X_{i}{\hat{\beta }}^{0}$ and then use $\hat{\sigma }={\text{Median}}_{1\leq i\leq n}\left|e_{i}\left({\hat{\beta }}^{0}\right)-{\text{Median}}_{1\leq i^{\prime }\leq n}e_{i^{\prime }}\left({\hat{\beta }}^{0}\right)\right|,$ which might give higher power than using the other estimate of *σ* (which does not take account of possible effects of RVs). However, it is difficult to obtain reliable estimates of ${\hat{\beta }}^{0}$ for RVs, which in fact motivated the development of the burden tests and other methods. This is a topic to be explored in the future.

### Comparison with Winsorizing and trimming

Two simple and straightforward ways to handle outliers are Winsorizing and trimming. For a specified small $\alpha_{1},$ such as $\alpha_{1}=0.05$ or 0.025, define the $100\times \alpha_{1}$-percentile and $100\times \alpha_{1}$-percentile of $\left\{Y_{1},Y_{2},\ldots ,Y_{n}\right\}$ as $y_{\alpha_{1}}$ and $y_{1-\alpha_{1}}$ respectively. In Winsorizing, any $Y_{i}$ satisfying $Y_{i}<y_{\alpha_{1}}$ is truncated at $y_{\alpha_{1}},$ and any $Y_{i}>y_{1-\alpha_{1}}$ is truncated at $y_{1-\alpha_{1}}.$ In trimming, any observation *i* is removed from the dataset if $Y_{i}<y_{\alpha_{1}}$ or $Y_{i}>y_{1-\alpha_{1}}.$

Winsorizing is to some degree like using the Huber loss function in truncating outlying trait values. However there are two important differences. First, the choice of the threshold $\alpha_{1}$ is arbitrary, which may be too small or too large, depending on the unknown proportion of the outliers. Second, more importantly, in Winsorizing whether a trait value $Y_{i}$ is judged to be an outlier or not is completely based on its absolute value $\left|Y_{i}\right|$ without accounting for any covariates; if instead we Winsorize residuals $\left|Y_{i}-Z_{i}\hat{\gamma }\right|,$ it will be more similar to using the Huber loss. As will be shown, ignoring covariate effects may lead to severely inflated type I errors or power loss.

In addition to the above two disadvantages shared with Winsorizing, trimming is too extreme in eliminating the observations judged to be, but in truth may or may not be, outliers, which often leads to severe loss of power.

### Software and data availability

The aSPUr test has been implemented in *R* package “aSPU” available on the Comprehensive R Archive Network (CRAN): https://cran.r-project.org/web/packages/aSPU/. The NHLBI ESP data are accessible from the National Center for Biotechnology Information (NCBI) dbGaP with accession numbers phs000398, phs000400, phs000401, and phs000281.

## Results

### Simulation set-ups

To evaluate and compare the performance of various tests, we conducted extensive simulation studies under different trait distributions. The genotype data were simulated following Wang and Elston (2007) and Basu and Pan (2011). Specifically, a latent variable $L_{1}={\left(L_{11},\ldots ,L_{1k}\right)}^{\prime }$ was simulated from a *k*-dimensional multivariate normal distribution N(0,V) with *V* as an AR-1(*ρ*) correlation structure: $V_{j,l}=\rho^{\left|j-l\right|}$ for any 1≤j,l≤k. Then we randomly drew from a uniform distribution U(0.001,0.005) *k* MAFs between 0.1 and 0.5%, and accordingly dichotomized $L_{i}$ to yield a haplotype. We similarly simulated another latent variable and the corresponding haplotype. We combined the two haplotypes to form the genotype $X_{i}$ for subject *i*. This process was repeated n=400 times to generate genotypes for n=400 subjects. We used ρ=0 and ρ=0.8 to generate independent and correlated SNVs (in linkage equilibrium and in linkage disequilibrium) respectively. Note that we only used unphased genotypes, not haplotypes, in simulations.

A trait $Y_{i}$ was simulated from linear regression model $Y_{i}=X_{i}\beta +Z_{i}\gamma +\epsilon_{i}$ with the following error distribution. First, $\epsilon_{i}$ was independent and identically distributed (iid) from ∼N(0,1). Second, $\epsilon_{i}$ was iid from $∼LN\left(0,\sigma_{e}\right),$ a Log-normal distribution with mean 0 and SD $\sigma_{e}$ on the log scale. Third, $\epsilon_{i}$ was iid from $∼t_{d},$ a *t*-distribution with degrees of freedom d=1 or 3. Fourth, $\epsilon_{i}$ was iid from a contaminated N(0,1): a single observation $i_{0}$ with $\sum_{j}X_{i_{0}j}>0$ was randomly chosen and its trait had an additive error $e∼N\left(0,\sigma_{e}\right)$ with $\sigma_{e}=5$ or 10.

To evaluate the empirical type I error (null cases), we had β=0. For empirical power (nonnull cases), we randomly chose eight SNVs from *k* SNVs as causal ones with nonzero $\beta_{j}$’s while other SNVs having their $\beta_{j}=0.$ For causal SNVs, we used two sets of coefficients: $\beta ={\left(-1.2,-1.2,-0.8,-0.8,0.8,1,1,1\right)}^{\prime }$ (power set-up I) and $\beta ={\left(0.7,0.7,0.7,1,1,1,1.2,1.2\right)}^{\prime }$ (power set-up II), which favors SKAT and the burden test, respectively. In the absence of other covariates, $Z_{i}$ was just a constant 1 for the intercept term; otherwise, we randomly generated two independent covariates from N(0,1) with $\gamma ={\left(1,-1\right)}^{\prime }.$ To investigate the robustness of a method to the number of SNVs, we increased *k* from 8 to 256.

### Simulation results

Table 1 shows the empirical type I error rates for various tests without any transformation. Under N(0,1) error distribution, all tests controlled the type I error rates satisfactorily at the nominal level α=0.05. Under heavy-tailed ($t_{3}$ and $t_{1}$) and skewed (LN(0,1) and LN(0,2)) error distributions, SKAT and SKAT-O had severely inflated type I error rates, while aSPU and aSPUr controlled their type I error rates satisfactorily. With even just 1 (out of 400) quantitative trait contaminated, all the tests except aSPUr could not control the type I error rates well, though the aSPU test (along with the SPU tests) performed much better than SKAT and SKAT-O. The same conclusions held with correlated SNVs and with or without covariates (Supplemental Material, Table S1, Table S2, and Table S3). For nonnormal error distributions subject to Winsorizing or trimming, the results were dependent on the choice of the cut-off $\alpha_{1}$ as shown in Table S4 and Table S5. For example, when the error distribution was $t_{1}$ with no covariates, SKAT and SKAT-O after Winsorizing at $\alpha_{1}=0.05$ could maintain a correct type I error rate, but not at $\alpha_{1}=0.025.$ For an error distribution of LN(0,2), SKAT and SKAT-O could not control the type I error rates for either $\alpha_{1}=0.05$ or $\alpha_{1}=0.025$ (Table S4). With covariates, the performance became worse, especially with trimming; neither Winsorizing nor trimming could control type I error rates at either $\alpha_{1}=0.05$ or $\alpha_{1}=0.025$ (Table S5).

**Table 1:** Empirical type I error rates of various tests at the significance level of 0.05 for a quantitative trait with an error distribution (Distr), a number of independent SNVs (#SNVs), and with two covariates

Distr	#SNVs	SKAT	SKAT-O	SPU(1)	SPU(2)	SPU(3)	SPU(4)	SPU(∞)	aSPU	aSPUr
N(0,1)	8	0.044	0.053	0.048	0.050	0.055	0.055	0.059	0.057	0.055
	32	0.064	0.058	0.065	0.063	0.051	0.061	0.056	0.063	0.056
	64	0.050	0.047	0.047	0.053	0.049	0.055	0.054	0.047	0.052
	128	0.044	0.041	0.049	0.052	0.051	0.053	0.047	0.047	0.053
	192	0.031	0.032	0.049	0.039	0.048	0.053	0.054	0.049	0.048
	256	0.019	0.031	0.051	0.025	0.040	0.035	0.037	0.041	0.033
$t_{3}$	8	0.076	0.072	0.051	0.042	0.047	0.043	0.047	0.048	0.046
	32	0.113	0.105	0.050	0.046	0.050	0.055	0.052	0.045	0.042
	64	0.132	0.109	0.039	0.034	0.039	0.049	0.051	0.040	0.047
	128	0.114	0.105	0.048	0.027	0.045	0.042	0.057	0.048	0.047
	192	0.104	0.101	0.065	0.019	0.032	0.032	0.050	0.044	0.048
	256	0.087	0.074	0.042	0.007	0.013	0.022	0.043	0.026	0.062
$t_{1}$	8	0.082	0.081	0.052	0.048	0.051	0.049	0.050	0.049	0.031
	32	0.190	0.186	0.060	0.064	0.051	0.062	0.078	0.061	0.040
	64	0.289	0.268	0.043	0.036	0.033	0.036	0.100	0.062	0.042
	128	0.310	0.276	0.038	0.025	0.027	0.028	0.085	0.050	0.033
	192	0.269	0.251	0.036	0.011	0.015	0.019	0.054	0.031	0.032
	256	0.310	0.282	0.036	0.006	0.013	0.016	0.064	0.037	0.036
LN(0,1)	8	0.107	0.093	0.056	0.065	0.063	0.062	0.061	0.067	0.053
	32	0.160	0.137	0.052	0.041	0.052	0.053	0.061	0.052	0.047
	64	0.165	0.144	0.052	0.038	0.037	0.043	0.057	0.045	0.038
	128	0.176	0.147	0.053	0.030	0.049	0.050	0.059	0.048	0.052
	192	0.173	0.142	0.045	0.012	0.029	0.035	0.049	0.033	0.050
	256	0.151	0.115	0.043	0.007	0.025	0.027	0.047	0.039	0.050
LN(0,2)	8	0.113	0.103	0.063	0.056	0.059	0.059	0.061	0.058	0.057
	32	0.209	0.197	0.043	0.043	0.058	0.060	0.075	0.058	0.051
	64	0.276	0.259	0.045	0.038	0.040	0.044	0.062	0.053	0.059
	128	0.277	0.251	0.052	0.032	0.039	0.044	0.069	0.045	0.064
	192	0.269	0.241	0.033	0.013	0.022	0.025	0.054	0.026	0.063
	256	0.287	0.249	0.035	0.010	0.019	0.023	0.051	0.034	0.056
N(0,1) contaminated $\sigma_{e}=5$	8	0.371	0.316	0.177	0.333	0.327	0.340	0.337	0.290	0.060
	32	0.226	0.187	0.078	0.139	0.136	0.143	0.155	0.121	0.054
	64	0.147	0.120	0.058	0.077	0.083	0.084	0.083	0.080	0.055
	128	0.089	0.089	0.061	0.048	0.054	0.054	0.069	0.068	0.060
	192	0.060	0.055	0.049	0.035	0.049	0.040	0.055	0.050	0.039
	256	0.045	0.041	0.041	0.027	0.045	0.035	0.060	0.040	0.047
N(0,1) contaminated $\sigma_{e}=10$	8	0.605	0.582	0.365	0.563	0.566	0.572	0.564	0.516	0.061
	32	0.477	0.444	0.118	0.201	0.209	0.211	0.230	0.174	0.054
	64	0.349	0.298	0.089	0.096	0.117	0.118	0.142	0.104	0.057
	128	0.178	0.155	0.067	0.043	0.056	0.054	0.086	0.064	0.060
	192	0.142	0.131	0.047	0.033	0.046	0.040	0.051	0.044	0.041
	256	0.112	0.099	0.040	0.020	0.043	0.034	0.068	0.037	0.048

For empirical power comparison, under N(0,1) error distribution the aSPU test performed similarly to SKAT or SKAT-O with a smaller number of SNVs; however, as the number of SNVs increased, the aSPU test became more powerful (Table S6). As reported before (Pan *et al.* 2014; 2015a,b), with increasing number of SNVs an SPU(*γ*) test with a larger γ>0 value tended to be more powerful; in particular, SPU(4) could be much more powerful than SPU(2), *e.g.*, when there were 128 or more SNVs (Table S6). Of note, SPU(2) is equivalent to SKAT with a linear kernel which was optimal here and was used throughout (Pan 2009; Pan 2011). On the other hand, the aSPUr was conservative, especially for causal SNVs with larger effect sizes (Cases III and IV in Table S6), in which it was hard to distinguish a genuinely large effect size of a RV from a contaminated trait value. In robust statistics, one would like to use some initial estimator to estimate and thus take account of large effects, which however was almost impossible in the current context for RVs: with small MAFs, it is almost impossible to obtain reliable estimates for RVs. The same conclusions held with correlated SNVs (Table S7). For Winsorizing or trimming, there was always a dramatic loss of power with trimming, while Winsorizing performed well for a smaller number of SNVs. But its performance deteriorated as the number of SNVs increased; in particular, again its performance depended on the use of the $\alpha_{1}$ level (Table S8 and Table S9).

We further investigated the effects of natural logarithm (Ln) and rank-based inverse normal (INV) transformations on the type I error rate and power. We considered the skewed LN(0,1) and heavy-tailed $t_{3}$ error distributions. With the two covariates in the simulated data, we first regressed them out in a linear model under $H_{0},$ then used the residuals or their transformations to test their association with a set of SNVs.

**Figure 1:**
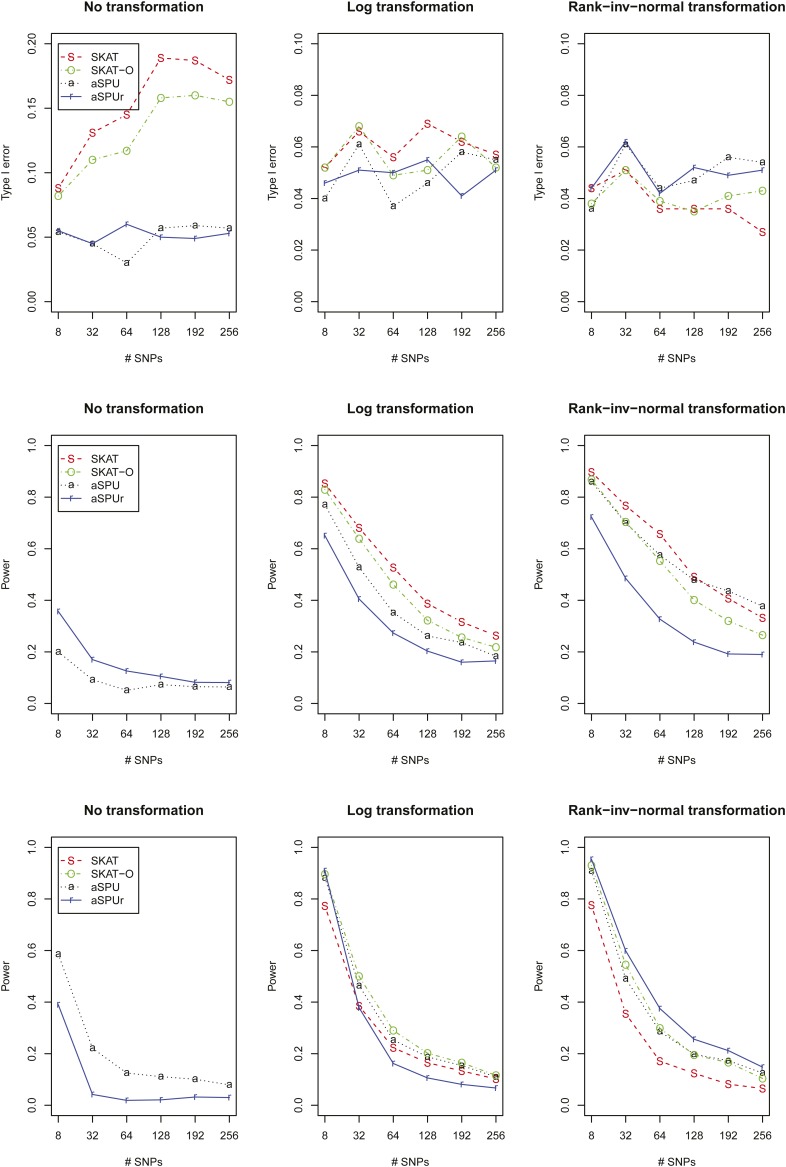
Simulation results for a skewed error distribution LN(0,1): the first row is for type I errors, and the next two rows for power in set-up I with $\beta ={\left(-1.2,-1.2,-0.8,-0.8,0.8,1,1,1\right)}^{\prime }$ and set-up II with $\beta ={\left(0.7,0.7,0.7,1,1,1,1.2,1.2\right)}^{\prime }.$

Figure 1 shows the type I error rates and powers for a skewed error distribution LN(0,1). First, without transformation both SKAT and SKAT-O gave severely inflated type I error rates, while aSPU and aSPUr controlled their type I error rates satisfactorily; with Ln transformation, all tests performed well, although the type I error rates of SKAT and SKAT-O might be slightly inflated; with INV transformation, all tests controlled the type I error rates satisfactorily. Second, under power set-up I which favored SKAT and SPU(2) because the association directions of the eight causal SNVs were different, without transformation although both aSPU and aSPUr could control the type I error rates, they lost power dramatically as compared to those with transformed traits; between the two, aSPUr was more powerful. On the other hand, with Ln transformation SKAT was most powerful, followed by SKAT-O, then aSPU, and finally aSPUr, though the power difference became smaller as the number of SNVs to be tested increased; with INV transformation, SKAT was most powerful for smaller numbers of SNVs while aSPU was more powerful for larger numbers of SNVs; for unknown reasons, aSPUr did not perform well. Third, under power set-up II which favored burden tests because the association directions of the eight causal SNVs were the same, without transformation although both aSPU and aSPUr could control the type I error rates, they lost power dramatically as compared to those with transformed traits; between the two, aSPU was more powerful. With Ln transformation, SKAT-O and aSPU were most powerful, followed by SKAT or aSPUr, though the power difference was not dramatic; with INV transformation, aSPUr was consistently best, followed by SKAT-O and aSPU (for which the former had an edge for a smaller number of SNVs while the latter had otherwise), finally by SKAT.

**Figure 2:**
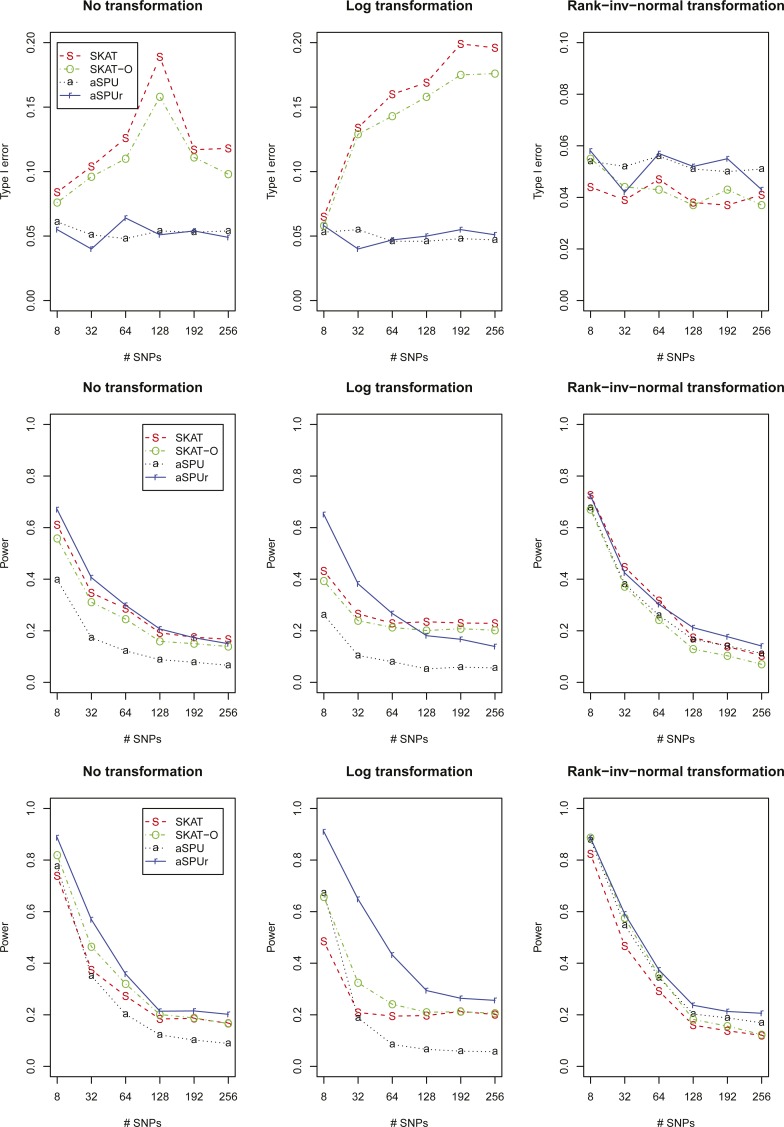
Simulation results for a heavy-tailed (and nonskewed) error distribution $t_{3}:$ the first row is for type I errors, and the next two rows for power in set-up I with $\beta ={\left(-1.2,-1.2,-0.8,-0.8,0.8,1,1,1\right)}^{\prime }$ and set-up II with $\beta ={\left(0.7,0.7,0.7,1,1,1,1.2,1.2\right)}^{\prime }.$

Figure 2 shows the type I error rates and powers for a heavy-tailed (and nonskewed) error distribution $t_{3}.$ First, without transformation both SKAT and SKAT-O gave severely inflated type I error rates, while aSPU and aSPUr controlled their type I error rates satisfactorily. Although no reason to use a Ln transformation, to show possible effects of using an incorrect transformation, we also presented results based on the Ln transformation: again both aSPU and aSPUr were robust with well-controlled type I error rates, while SKAT and SKAT-O had severely inflated ones; with INV transformation, all tests were satisfactory. On the other hand, under power set-up I, with no or Ln transformation, although both aSPU and aSPUr could control the type I error rates, aSPU lost power dramatically while aSPUr did not as compared to those with transformed traits; between the two, aSPUr was much more powerful. We showed the results for SKAT and SKAT-O, even though they had severely inflated type I error rates. With INV transformation, SKAT and aSPUr were the winners, though SKAT was slightly more powerful for smaller numbers of SNVs while aSPUr was more powerful for larger numbers of SNVs, closely followed by aSPU, then SKAT-O. Finally, under power set-up II, with no or Ln transformation, although both aSPU and aSPUr could control the type I error rates, aSPU lost power dramatically while aSPUr did not as compared to those with transformed traits; between the two, aSPUr was more powerful; with INV transformation, aSPUr was best, closely followed by aSPU, then SKAT-O, and then SKAT.

### Data example: application to the NHLBI ESP triglyceride phenotype

To further demonstrate the performance of various RV tests in a real data example, we analyzed the WES data in association with plasma triglyceride level in 1731 individuals of European ancestry who were sequenced in the NHLBI ESP project. The study subjects were selected from the following population-based cohorts: Atherosclerosis Risk in Communities, the Cardiovascular Heart Study, the Framingham Heart Study, and the Womens Health Initiative; see Crosby *et al.* (2014) for details. We performed gene-based RV association tests, including SKAT, SKAT-O, T1 burden test, aSPU, and aSPUr, on untransformed, natural logarithm transformed, and rank-based inverse normal transformed triglyceride levels, denoted as TG, Ln(TG), and INV(TG), respectively. Following Crosby *et al.* (2014), we included nonsynonymous (nonsense and missense) and splice-site variants of MAF ≤1% within each gene and excluded genes with cumulative MACs <5, resulting in 13,978 genes. The genome-wide significance threshold was set at $3.6\times 10^{-6}$ based on the Bonferroni procedure. As in Crosby *et al.* (2014), we performed natural logarithm transformation on the raw triglyceride level and adjusted for covariates including age, sex, two principal components capturing population substructure, and indicator variables for the ESP ascertainment scheme in all association testing. We used QQ plots and GC *λ* to detect possible inflation of the RV association test p-values. Because there were a large number of extremely rare variants, *e.g.*, singletons and doubletons, in the ESP WES data, we used the power set Γ={1,2,3,4,5,6} for both aSPU and aSPUr, as suggested by Pan *et al.* (2014) for numerical stability. In addition, we used the following stage-wise bootstrap procedure for aSPU and aSPUr: we started with B=1000 for all genes and then gradually increased *B*. If an estimated p-value was <50/B, we increased *B* to 10×B to reestimate the p-value until $B=10^{6}$ for genome-wide significance. Moreover, we used *APOC3* as a positive control gene to compare the power of various tests. *APOC3* was identified as the top gene harboring putatively functional RVs associated with reduced level of Ln(TG), and was further replicated and confirmed in independent large samples (Crosby *et al.* 2014). In addition, it was identified in other RV association studies (Tachmazidou *et al.* 2013; Li *et al.* 2015).

**Figure 3:**
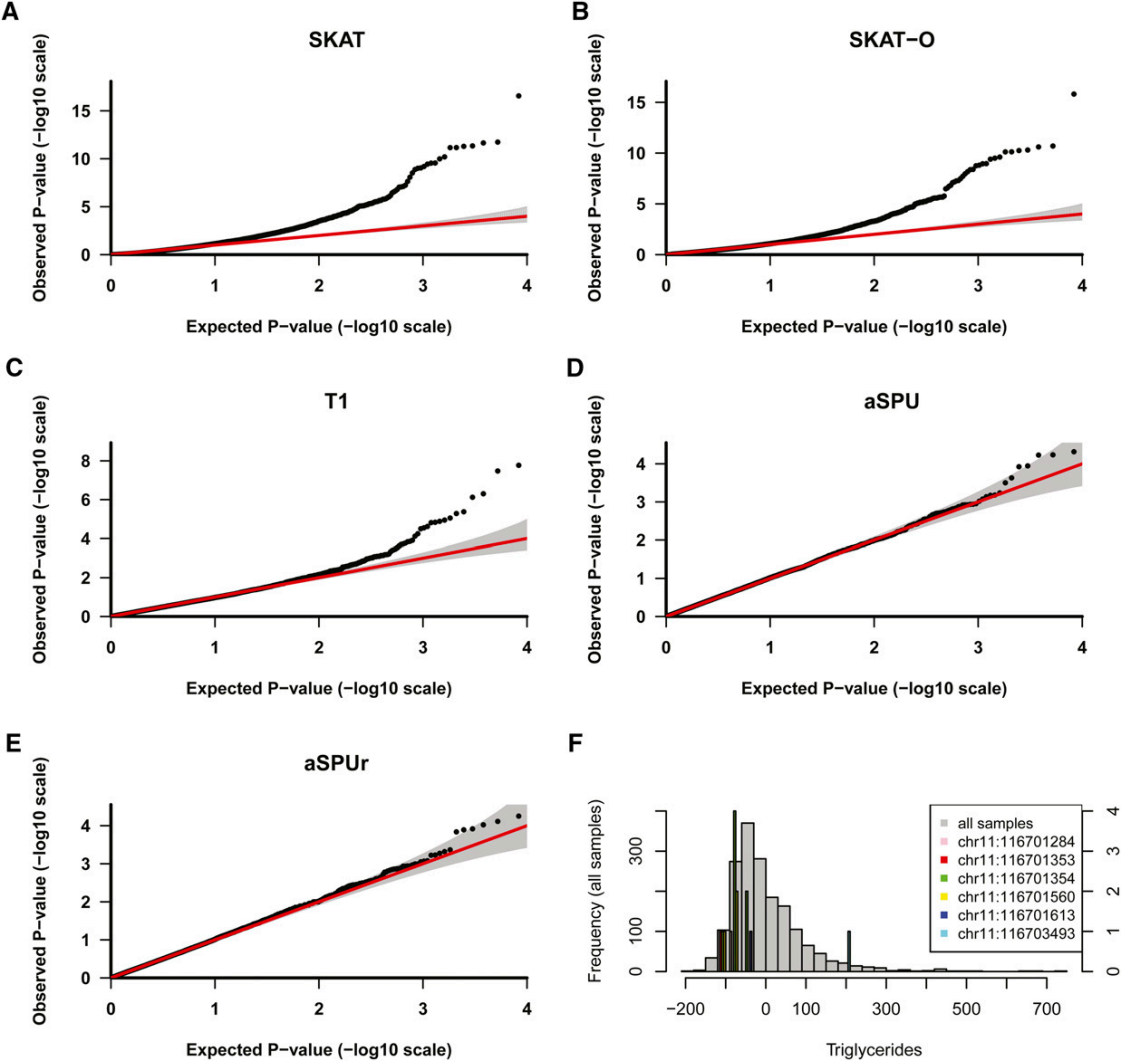
QQ plots for the analysis of triglyceride with 13,978 genes with MAC ≥5. (A) SKAT (genomic control λ=1.89), (B) SKAT-O (λ=1.78), (C) T1 (λ=1.13), (D) aSPU (λ=1.02), and (E) aSPUr (λ=1.04). (F) Histogram of covariate-adjusted triglyceride residuals with variant carriers of *APOC3* highlighted.

Figure 3F shows that TG was right-skewed with some individuals having extremely high TG levels. When applied to TG, the QQ plots for aSPU and aSPUr behaved normally as shown in Figure 3 (λ<1.04). In contrast, the SKAT and SKAT-O tests had severely inflated QQ plots (*λ* = 1.89 and 1.78, respectively); the QQ plot for T1 was less inflated but had a discernable deviation from the null in the tail area (*λ* = 1.13). We investigated the effectiveness of some *ad hoc* strategies, including trimming, Winsorizing, and increasing the MAC threshold, in alleviating the p-value inflation. When trimming at $\alpha_{1}=2.5\text{\%,}$ *λ* for SKAT and SKAT-O was reduced to 1.09 and 1.10, respectively; when Winsorizing at $\alpha_{1}=2.5\text{\%,}$ *λ* was reduced to 1.12 and 1.11, respectively. Despite the improvement, the QQ plots for SKAT and SKAT-O remained inflated (Figure S1). When we excluded genes with a MAC <30, the QQ plots for SKAT and SKAT-O were still inflated with *λ* = 1.36 and 1.30, respectively, whereas T1 had a much improved QQ plot (*λ* = 1.04) (Figure S2). However, increasing the MAC threshold to 30 would further exclude 8155 genes, including the positive control gene *APOC3* with 14 minor alleles. When applied to Ln(TG) and INV(TG), all tests had well-behaved QQ plots and $\lambda^{\prime }s$ <1.06 (Figure S3 and Figure S4). As shown in Figure 3F, TG approximately followed a Log-normal distribution, leading to similar results from the Ln and INV transformations; see the p-value comparison for *APOC3* in Table 2. To investigate whether the RV association test p-value inflation was also applicable to common variants, we performed conventional variant-by-variant association testing of TG for 50,602 SNPs with an MAF ≥5%. As shown in Figure S5, the QQ plot was well behaved with λ=1.02, suggesting that the p-value inflation was likely a unique problem for some RV association tests.

**Table 2:** RV association testing results of positive control gene *APOC3* (among 13,978 genes with a MAC ≥5)

Phenotype		SKAT	SKAT-O	T1	aSPU	aSPUr
TG	GC *λ*	1.89	1.78	1.13	1.02	1.04
	*APOC3* p-value	0.018	0.021	0.018	0.035	0.0036
	*APOC3* rank	642	620	297	501	62
Ln(TG)	GC *λ*	1.05	1.06	1.03	1.01	1.03
	*APOC3* p-value	$2.27\times 10^{-4}$	$3.70\times 10^{-5}$	$4.19\times 10^{-5}$	$1.18\times 10^{-4}$	$3.30\times 10^{-5}$
	*APOC3* rank	6	1	1	2	1
INV(TG)	GC *λ*	1.03	1.05	1.03	1.02	1.04
	*APOC3* p-value	$2\times 10^{-4}$	$3.85\times 10^{-5}$	$4.67\times 10^{-5}$	$9.70\times 10^{-5}$	$3.30\times 10^{-5}$
	*APOC3* rank	6	1	1	2	2

Table 2 shows the p-values and ranking of *APOC3* by various tests. In the analysis of TG, *APOC3* was ranked 62nd by aSPUr, but was not among the top 200 genes by all other methods; in the analyses of Ln(TG) and INV(TG), it was ranked among the top two by T1, SKAT-O, aSPU, and aSPUr, but not SKAT. This is consistent with the results reported in Crosby *et al.* (2014) that *APOC3* was the top gene associated with Ln(TG) by the T1 test but its p-value was not genome-wide significant in the discovery samples from the ESP. We demonstrate here that the statistical significance of *APOC3* was dependent on the transformation of the phenotype TG. Figure 3F shows that the carriers of the minor alleles for five out of six RVs in *APOC3* had reduced TG levels compared with the population average. In the presence of quite a few individuals with extremely high TG levels, *APOC3* was only nominally associated with TG and lowly ranked by all RV association tests except for aSPUr. By downweighting the extremely high TG observations, aSPUr increased the statistical significance and ranking of *APOC3* compared with aSPU and other tests, while avoiding global inflation of the p-values. On the other hand, since both Ln and INV transformations reduced the impact of extremely high TG observations, the association signal of *APOC3* was much amplified, resulting in its high ranking. In addition, as the majority of the variants in *APOC3* reduced the TG level, *i.e.*, the effects were roughly in the same direction, the T1 burden test and adaptive tests that incorporate the burden test, such as aSPU, aSPUr, and SKAT-O, yielded higher ranking for *APOC3* than did SKAT.

## Discussion

In summary, we have demonstrated using extensive simulations and application to the ESP WES data that SKAT and SKAT-O are not robust to heavy-tailed or skewed error distributions of quantitative traits with inflated type I error rates. *Ad hoc* remediation procedures, such as trimming and Winsorizing, may not be effective in reducing the inflated type I error rates and may lead to severe power loss. Depending on the underlying trait distributions, Ln or INV transformation may help control the type I error rates for SKAT and SKAT-O, which, however, could lead to transformation-specific association results as illustrated in the *APOC3* example, as well as power loss as demonstrated in simulation set-up II in Figure 2. On the other hand, the aSPU test and the newly proposed aSPUr test are much more robust to quantitative traits’ deviation from normality.

The nonrobustness of the SKAT test is mainly due to its poor asymptotic approximation of the null distribution in the presence of outliers. Note that the issue with SKAT remained with the use of its resampling method to calculate its p-values: we found that SKAT-Resampling implemented in the *R* package “SKAT” gave essentially equal p-values to those of SKAT in both simulations and real data application; the Pearson correlation between the two sets of the p-values was >0.999. Moreover, the RV weighting scheme of SKAT makes its type I error inflation even worse. Since SKAT puts a higher weight on a more rare SNV *j*, if $X_{ij}=1,$ then it is a high-leverage point; in addition, if $Y_{i}$ is outlying, then we know $\left(X_{ij},Y_{i}\right)$ is an influential point. Hence, although SKAT’s weighting on rare SNVs might help it gain power to detect associated RVs, at the same time, the weighting also renders its nonrobustness to observations with outlying traits, which could happen when the trait has a heavy-tailed or right-skewed distribution, as shown in our simulations and supported by the real data application. For the latter, when applied to TG, SKAT with its default Beta(1,25) weighting and equal weighting gave a GC *λ* of 1.89 and 1.86, respectively.

Recently Auer *et al.* (2016) also reported that single SNV-based and SNV-set-based RV tests can be nonrobust to phenotypic outliers and nonnormality, which is in agreement with the main theme of this paper and highlights the importance of the topic studied here. They recommended the INV transformation for nonnormally distributed traits. Our work here is distinctive from Auer *et al.* in several important aspects. First, in addition to demonstrating the nonrobustness of existing RV tests, we have proposed a new SNV-set-based robust RV test, aSPUr. Second, while Auer *et al.* applied the Huber robust regression in the context of single SNV-based RV testing, we impose the Huber loss on the score vector *U* in the proposed aSPUr, which is generalizable to the broad class of score vector-based SNV-set RV tests, *e.g.*, SPU(1)/T1 and SPU(2)/SKAT. Third, in contrast to the finding of Auer *et al.* that the permutation test was the least powerful method when applied to single SNV-based RV test, we found the permutation-based aSPU and aSPUr to be robust in terms of both type I error control and maintaining high statistical power in the presence of true signals. Finally, we found that while the INV transformation could maintain the type I error rate, it could also lead to transformation-dependent ranking order of p-values as demonstrated in the *APOC3* example (Table 2).

We proposed the aSPUr test in the Huber loss framework. It is one of the first proposed and most thoroughly studied loss function in the robust statistics literature. For example, when the error distribution is normal, it has been shown that the Huber loss achieves 95% asymptotic efficiency with the tuning parameter *c* being equal to 1.345 (Huber 1964). We found that it performed satisfactorily in our extensive numerical experiments. Other loss functions are also possible, for example, Tukeys biweight function (Jureckova and Picek 2006), which warrants further investigation.

In conclusion, we would recommend the use of the aSPU test for its robustness to heavily-tailed or skewed error distributions and its high power across many situations due to its adaptiveness. If there is evidence of inflated type I error or *λ*, *e.g.*, through QQ plots, then one may try the more robust aSPUr test or SKAT and SKAT-O with the INV transformation. Finally, neither Winsorizing nor trimming the data before applying another test, *e.g.*, SKAT or SKAT-O, outperformed the aSPUr test that was applied to the original data.

## Supplementary Material

Supplemental Material
