# Supplementary material for: On Robust Association Testing for Quantitative Traits and Rare Variants
Source: G3 (Bethesda). 2016 Sep 27;6(12):3941–50. doi: 10.1534/g3.116.035485 (PMC5144964; doi:10.1534/g3.116.035485)
Supplement: Supplemental Material [file supp_6_12_3941__index.html]

On Robust Association Testing for Quantitative Traits and Rare Variants — Supplemental Material 

# On Robust Association Testing for Quantitative Traits and Rare Variants

## Supplemental Material for Wei *et al.*, 2016

**Files in this Data Supplement:**

- Table S1 - Empirical type I error rates of various tests at the significance level of 0.05 for a quantitative trait with an error distribution (Distr) and a number of independent SNVs (#SNVs). (.pdf, 69 KB)
- Figure S1 - QQ plots for the analysis of triglyceride for 13978 genes with MAC ≥ 5. (.pdf, 495 KB)
- Figure S2 - QQ plots for the analysis of triglyceride for 13978 genes with MAC ≥ 5 (left panel) and for 5823 genes with MAC ≥ 30 (right panel). (.pdf, 391 KB)
- Figure S3 - QQ plots for the analysis of natural logarithm transformed triglyceride (Ln(TG)) with 13978 genes with MAC ≥ 5. (.pdf, 452 KB)
- Figure S4 - QQ plots for the analysis of rank-based inverse normal transformed triglyceride (INV(TG)) with 13978 genes with MAC ≥ 5. (.pdf, 446 KB)
- Figure S5 - QQ plot for the analysis of triglyceride (TG) with 50602 SNVs with minor allele frequency (MAF) ≥ 5%. (.pdf, 249 KB)
- Table S2 - Empirical type I error rates of various tests at the significance level of 0.05 for a quantitative trait with an error distribution (Distr) and a number of correlated SNVs (#SNVs). (.pdf, 69 KB)
- Table S3 - Empirical type I error rates of various tests at the significance level of 0.05 for a quantitative trait with an error distribution (Distr), a number of correlated SNVs (#SNVs) and with two covariates. (.pdf, 69 KB)
- Table S4 - Empirical type I error rates of various tests at the significance level of 0.05 after winsorizing or trimming (at level α1 = 0:05 or 0.025) a quantitative trait with an error distribution (Distr). (.pdf, 62 KB)
- Table S5 - Empirical type I error rates of various tests at the significance level of 0.05 after winsorizing or trimming (at level α1 = 0:05 or 0.025) a quantitative trait with an error distribution (Distr). (.pdf, 62 KB)
- Table S6 - Empirical power of various tests at the significance level of 0.05 for a quantitative trait and a number of independent SNVs (#SNVs), without any covariates. (.pdf, 61 KB)
- Table S7 - Empirical power of various tests at the significance level of 0.05 for a quantitative trait and a number of correlated SNVs (#SNVs), with two covariates. (.pdf, 60 KB)
- Table S8 - Empirical power of various tests at the significance level of 0.05 after winsorizing or trimming (at level α1 = 0:05 or 0.025) a quantitative trait. (.pdf, 68 KB)
- Table S9 - Empirical power of various tests at the significance level of 0.05 after winsorizing or trimming (at level α1 = 0:05 or 0.025) a quantitative trait. (.pdf, 68 KB)
