# Supplementary material for: On Robust Association Testing for Quantitative Traits and Rare Variants
Source: G3 (Bethesda). 2016 Sep 27;6(12):3941–50. doi: 10.1534/g3.116.035485 (PMC5144964; doi:10.1534/g3.116.035485)
Supplement: Supplemental Material [file supp_g3.116.035485_TableS1.pdf]

Table S1: Empirical type I error rates of various tests at the significance level of 0.05 for a quantitative trait with an error distribution (Distr) and a number of independent SNVs (#SNVs). One observation was contaminated with an additive error drawn from $N(0, \sigma^2)$ in the last two scenarios. There are NO covariates.

Distr	#SNVs	SKAT	SKAT-O	SPU(1)	SPU(2)	SPU(3)	SPU(4)	SPU(∞)	aSPU	aSPUr
$N(0,1)$	8	0.054	0.059	0.056	0.055	0.050	0.056	0.048	0.061	0.058
	32	0.045	0.048	0.050	0.044	0.039	0.046	0.038	0.055	0.038
	64	0.037	0.040	0.042	0.045	0.053	0.053	0.050	0.048	0.056
	128	0.031	0.037	0.048	0.046	0.058	0.042	0.058	0.046	0.049
	192	0.030	0.033	0.039	0.068	0.049	0.058	0.046	0.046	0.042
	256	0.022	0.034	0.041	0.050	0.051	0.061	0.060	0.049	0.045
t_3	8	0.090	0.074	0.047	0.051	0.051	0.054	0.055	0.048	0.056
	32	0.124	0.110	0.047	0.041	0.041	0.034	0.033	0.037	0.042
	64	0.119	0.105	0.043	0.036	0.036	0.053	0.056	0.051	0.050
	128	0.126	0.111	0.037	0.059	0.053	0.054	0.060	0.057	0.041
	192	0.116	0.107	0.046	0.041	0.045	0.036	0.039	0.041	0.054
	256	0.116	0.103	0.056	0.048	0.054	0.052	0.055	0.051	0.051
t_1	8	0.083	0.076	0.050	0.048	0.050	0.050	0.050	0.047	0.042
	32	0.210	0.188	0.053	0.059	0.060	0.060	0.056	0.061	0.047
	64	0.281	0.263	0.048	0.048	0.052	0.045	0.043	0.046	0.061
	128	0.301	0.263	0.049	0.040	0.046	0.044	0.053	0.058	0.049
	192	0.288	0.277	0.041	0.045	0.041	0.042	0.052	0.056	0.055
	256	0.307	0.278	0.043	0.049	0.046	0.057	0.059	0.058	0.046
$LN(0,1)$	8	0.099	0.083	0.046	0.048	0.049	0.048	0.050	0.055	0.041
	32	0.166	0.145	0.051	0.048	0.052	0.057	0.052	0.056	0.049
	64	0.169	0.138	0.042	0.048	0.051	0.056	0.057	0.052	0.051
	128	0.154	0.136	0.051	0.055	0.053	0.045	0.047	0.045	0.054
	192	0.177	0.142	0.057	0.056	0.051	0.046	0.039	0.043	0.045
	256	0.149	0.118	0.050	0.037	0.041	0.045	0.045	0.049	0.057
$LN(0,2)$	8	0.106	0.097	0.047	0.045	0.047	0.048	0.047	0.048	0.037
	32	0.206	0.194	0.049	0.053	0.052	0.058	0.054	0.055	0.054
	64	0.259	0.239	0.052	0.053	0.057	0.054	0.055	0.058	0.050
	128	0.259	0.217	0.050	0.047	0.046	0.045	0.047	0.051	0.067
	192	0.277	0.245	0.054	0.060	0.054	0.047	0.035	0.048	0.042
	256	0.264	0.231	0.039	0.043	0.040	0.039	0.048	0.046	0.062
$N(0,1)$	8	0.343	0.315	0.176	0.306	0.316	0.322	0.327	0.283	0.059
contaminated	32	0.204	0.185	0.086	0.123	0.126	0.131	0.128	0.124	0.057
$\sigma_e = 5$	64	0.141	0.117	0.062	0.090	0.089	0.088	0.078	0.081	0.063
	128	0.077	0.071	0.054	0.063	0.059	0.064	0.059	0.058	0.036
	192	0.058	0.072	0.065	0.065	0.063	0.063	0.057	0.066	0.056
	256	0.043	0.047	0.049	0.048	0.064	0.050	0.048	0.045	0.040
$N(0,1)$	8	0.607	0.582	0.365	0.558	0.568	0.574	0.576	0.506	0.057
contaminated	32	0.488	0.453	0.122	0.207	0.201	0.199	0.189	0.181	0.059
$\sigma_e = 10$	64	0.339	0.294	0.072	0.123	0.114	0.117	0.110	0.107	0.062
	128	0.193	0.164	0.056	0.080	0.072	0.075	0.070	0.071	0.033
	192	0.161	0.143	0.055	0.069	0.063	0.073	0.054	0.062	0.050
	256	0.121	0.113	0.047	0.048	0.061	0.061	0.057	0.058	0.043
