# Supplementary material for: On Robust Association Testing for Quantitative Traits and Rare Variants
Source: G3 (Bethesda). 2016 Sep 27;6(12):3941–50. doi: 10.1534/g3.116.035485 (PMC5144964; doi:10.1534/g3.116.035485)

Figure 1: QQ plots for the analysis of triglyceride with 13978 genes with MAC ≥ 5. Trimming (left panel) and Winsorizing (right panel) at $\alpha_1 = 2.5\%$. Genomic control (GC) λ is shown in the parentheses.

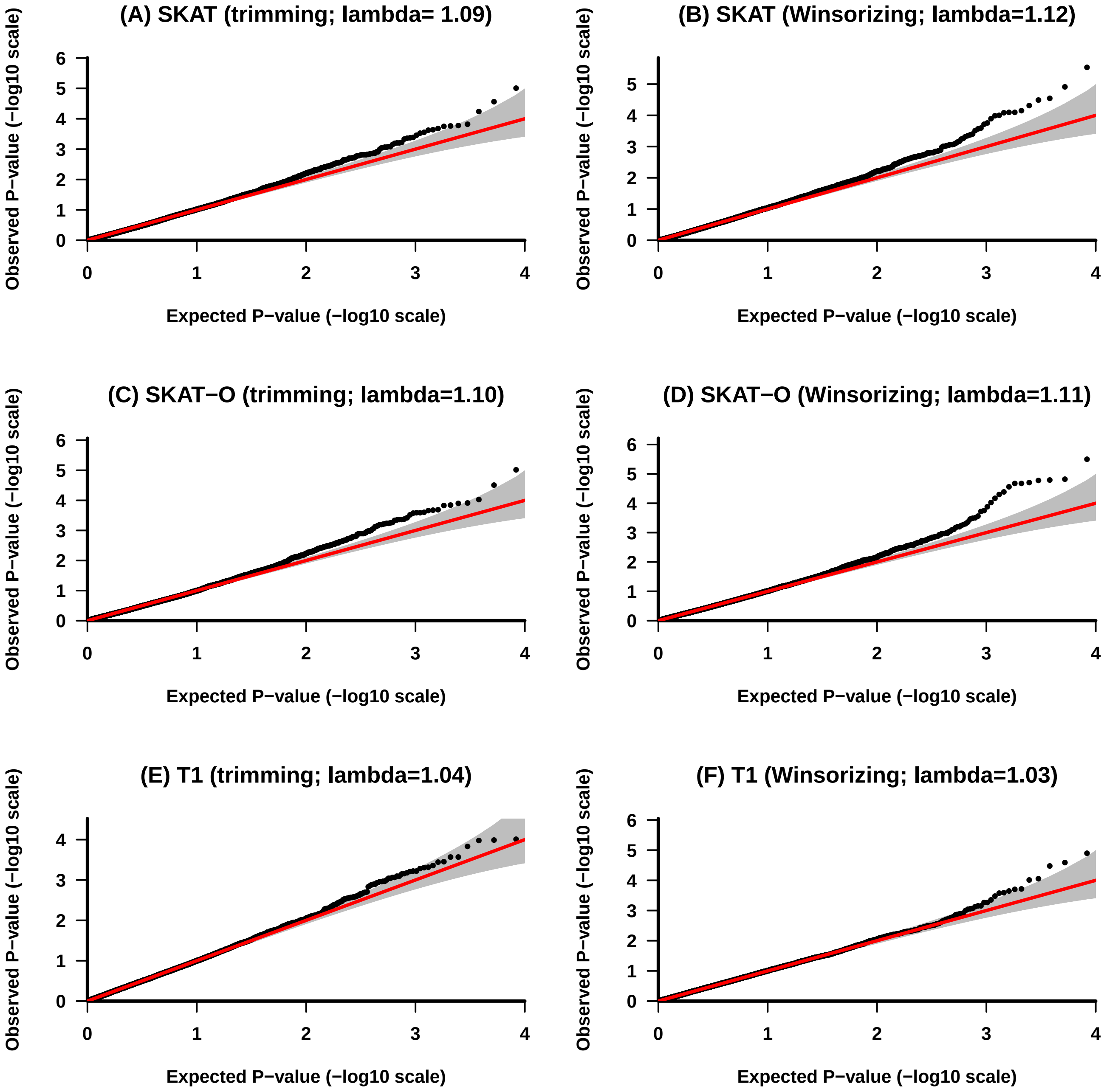

Supplement: Supplemental Material [file supp_g3.116.035485_FigureS1.pdf]
