# Supplementary material for: On Robust Association Testing for Quantitative Traits and Rare Variants
Source: G3 (Bethesda). 2016 Sep 27;6(12):3941–50. doi: 10.1534/g3.116.035485 (PMC5144964; doi:10.1534/g3.116.035485)

Figure 2: QQ plots for the analysis of triglyceride for 13978 genes with MAC ≥ 5 (left panel) and for 5823 genes with MAC ≥ 30 (right panel). GC λ is shown in the parentheses.

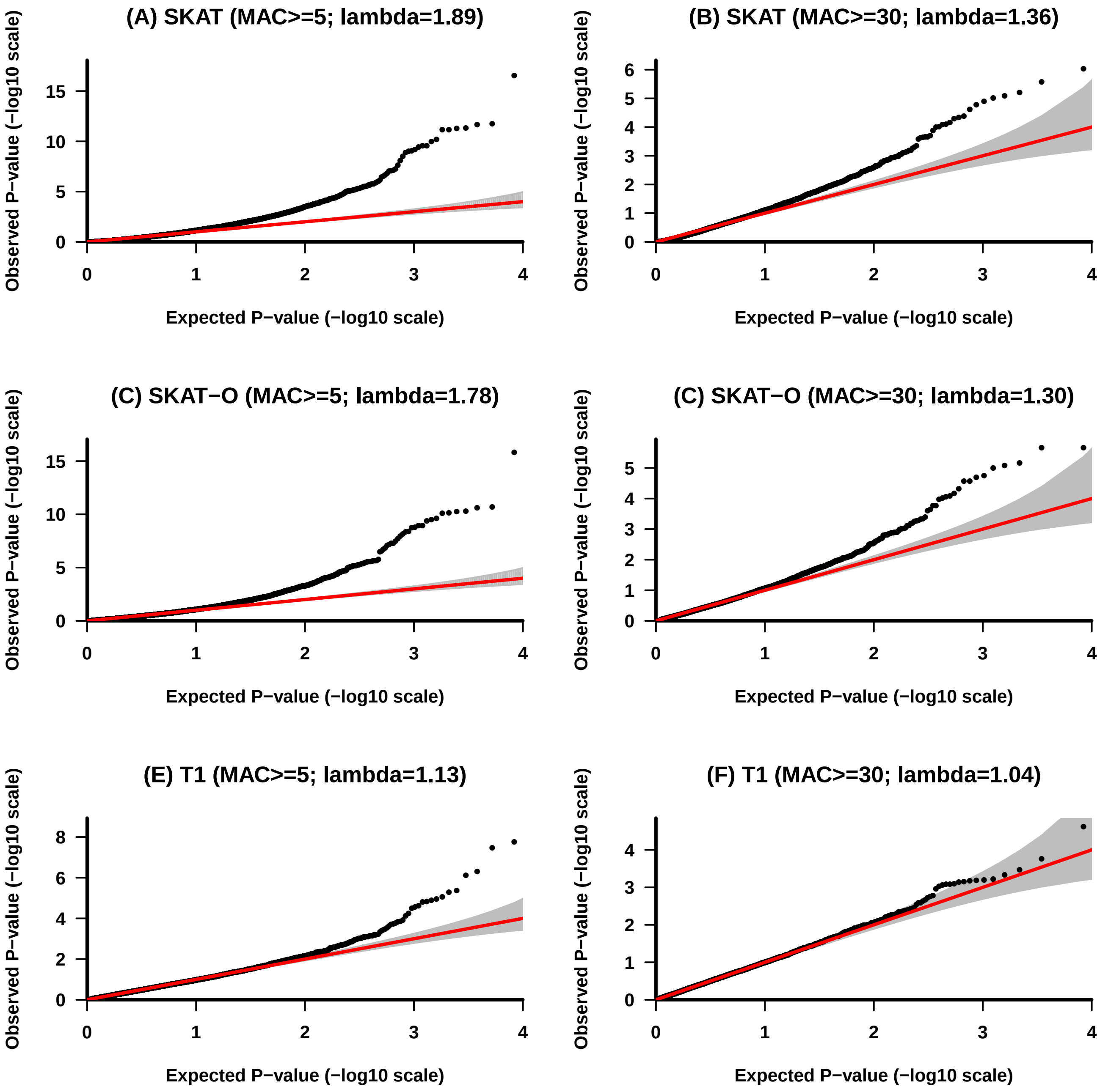

Supplement: Supplemental Material [file supp_g3.116.035485_FigureS2.pdf]
