# Supplementary material for: On Robust Association Testing for Quantitative Traits and Rare Variants
Source: G3 (Bethesda). 2016 Sep 27;6(12):3941–50. doi: 10.1534/g3.116.035485 (PMC5144964; doi:10.1534/g3.116.035485)

Figure 4: QQ plots for the analysis of rank-based inverse normal transformed triglyceride (INV(TG)) with 13978 genes with MAC ≥ 5. GC λ is shown in the parentheses. Panel (F): histogram of covariate-adjusted INV(TG) residuals with variant carriers of *APOC3* highlighted.

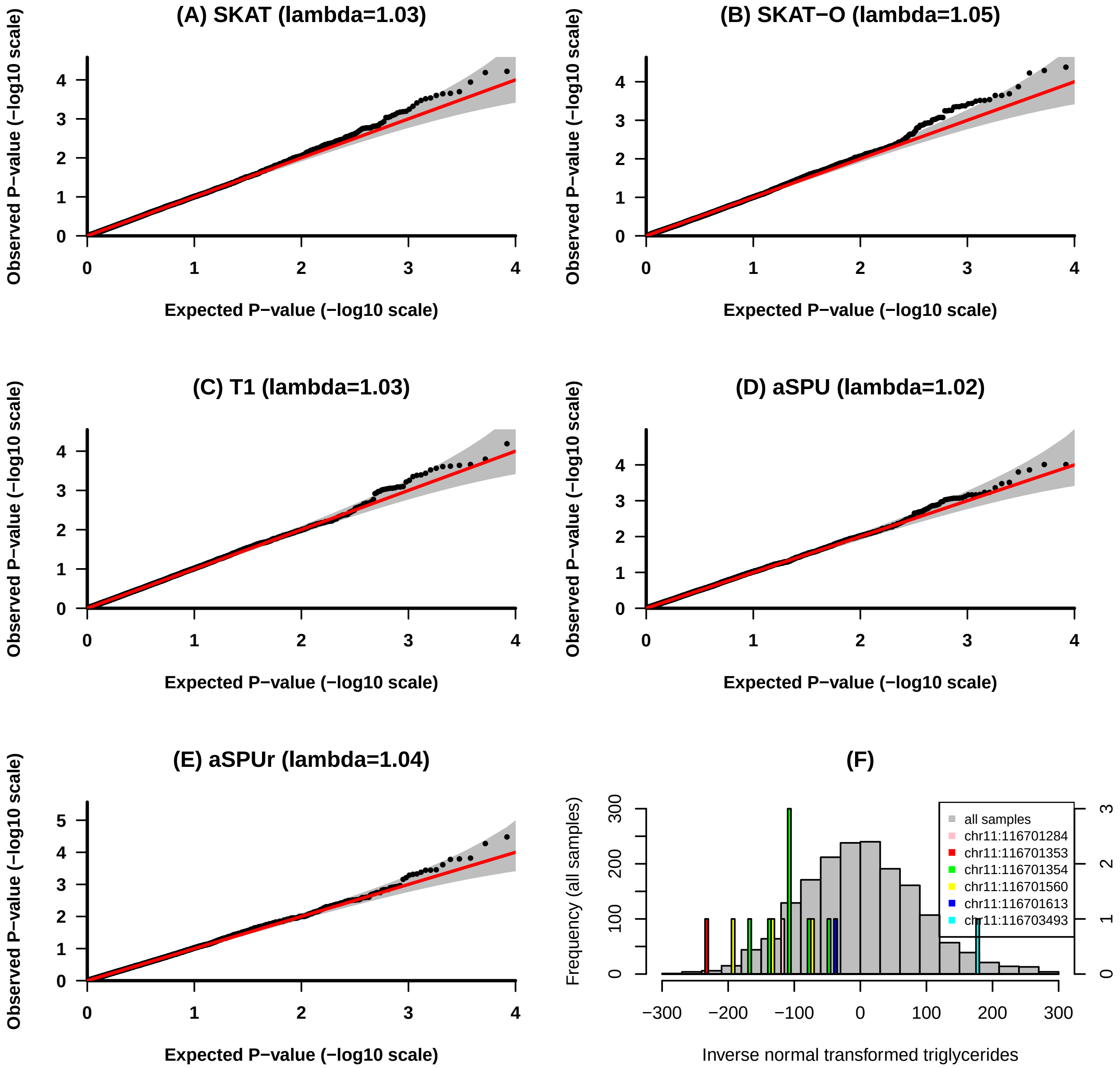

Supplement: Supplemental Material [file supp_g3.116.035485_FigureS4.pdf]
