# Supplementary material for: On Robust Association Testing for Quantitative Traits and Rare Variants
Source: G3 (Bethesda). 2016 Sep 27;6(12):3941–50. doi: 10.1534/g3.116.035485 (PMC5144964; doi:10.1534/g3.116.035485)

Figure 5: QQ plot for the analysis of triglyceride (TG) with 50602 SNVs with minor allele frequency (MAF) $\geq 5\%$.

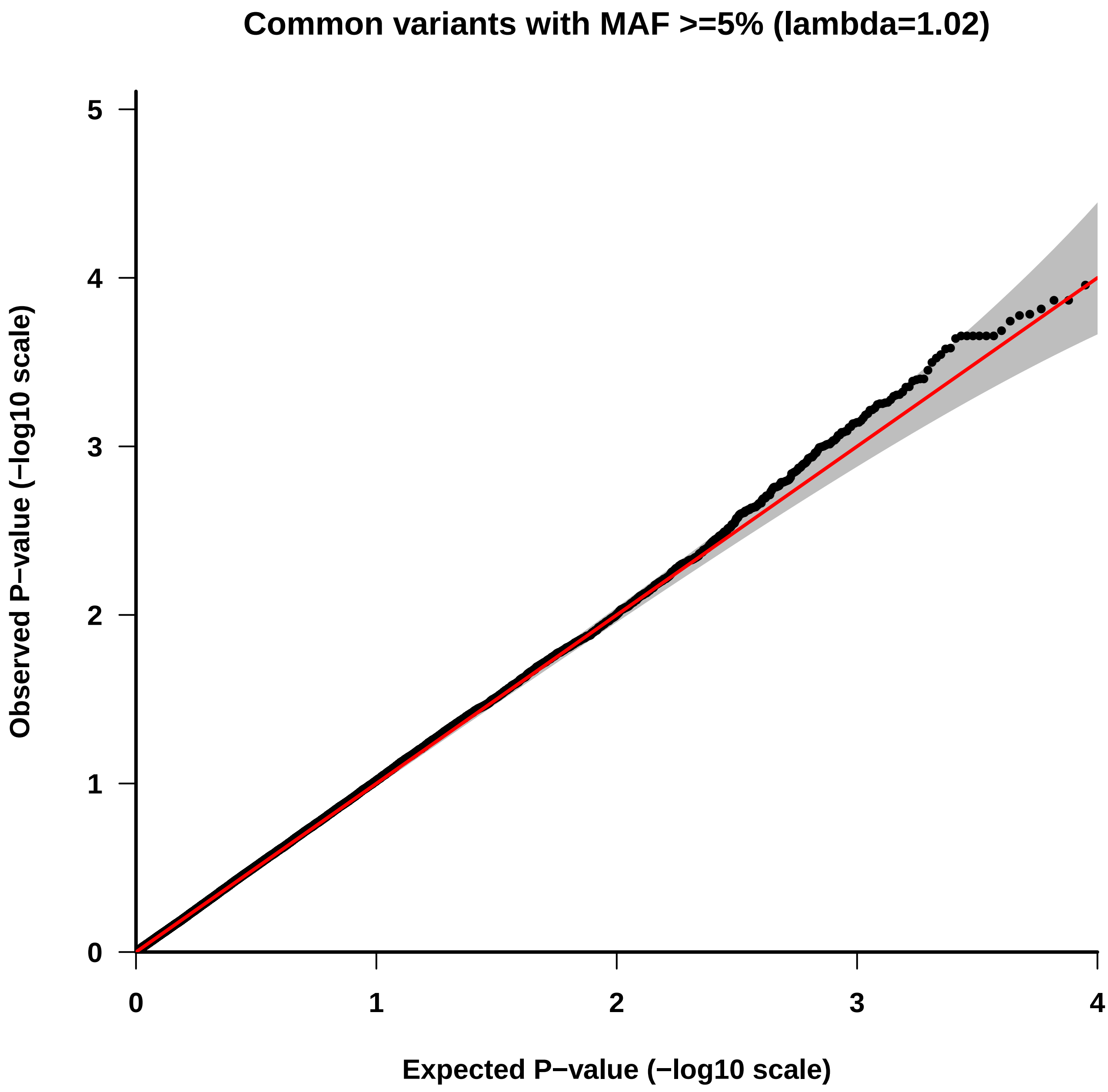

Supplement: Supplemental Material [file supp_g3.116.035485_FigureS5.pdf]
