# Supplementary material for: On Robust Association Testing for Quantitative Traits and Rare Variants
Source: G3 (Bethesda). 2016 Sep 27;6(12):3941–50. doi: 10.1534/g3.116.035485 (PMC5144964; doi:10.1534/g3.116.035485)
Supplement: Supplemental Material [file supp_g3.116.035485_TableS2.pdf]

Table S2: Empirical type I error rates of various tests at the significance level of 0.05 for a quantitative trait with an error distribution (Distr) and a number of correlated SNVs (#SNVs).

Distr	#SNVs	SKAT	SKAT-O	SPU(1)	SPU(2)	SPU(3)	SPU(4)	SPU(∞)	aSPU	aSPUr
$N(0,1)$	8	0.045	0.045	0.048	0.043	0.038	0.036	0.032	0.041	0.047
	32	0.044	0.051	0.051	0.046	0.047	0.051	0.046	0.045	0.051
	64	0.040	0.051	0.055	0.045	0.048	0.044	0.042	0.044	0.049
	128	0.035	0.046	0.054	0.047	0.044	0.047	0.042	0.044	0.050
	192	0.040	0.047	0.040	0.052	0.052	0.047	0.046	0.051	0.046
	256	0.031	0.044	0.056	0.058	0.050	0.054	0.043	0.053	0.058
t_3	8	0.069	0.060	0.043	0.044	0.047	0.047	0.052	0.050	0.045
	32	0.109	0.101	0.049	0.057	0.059	0.049	0.052	0.058	0.058
	64	0.121	0.102	0.043	0.044	0.042	0.045	0.047	0.046	0.050
	128	0.111	0.100	0.048	0.036	0.042	0.043	0.047	0.050	0.048
	192	0.141	0.122	0.057	0.066	0.059	0.054	0.053	0.053	0.064
	256	0.125	0.108	0.056	0.058	0.055	0.054	0.042	0.051	0.047
t_1	8	0.086	0.085	0.059	0.054	0.055	0.055	0.052	0.057	0.047
	32	0.175	0.166	0.050	0.045	0.044	0.045	0.062	0.060	0.039
	64	0.211	0.198	0.041	0.036	0.040	0.040	0.048	0.039	0.037
	128	0.230	0.215	0.052	0.048	0.045	0.048	0.047	0.045	0.051
	192	0.267	0.251	0.050	0.053	0.050	0.054	0.055	0.050	0.056
	256	0.285	0.257	0.039	0.045	0.044	0.046	0.057	0.048	0.053
$LN(0,1)$	8	0.108	0.094	0.053	0.060	0.056	0.056	0.058	0.072	0.058
	32	0.140	0.118	0.049	0.058	0.055	0.054	0.055	0.054	0.052
	64	0.139	0.121	0.037	0.038	0.039	0.039	0.044	0.043	0.044
	128	0.176	0.164	0.055	0.060	0.057	0.056	0.047	0.061	0.046
	192	0.191	0.169	0.055	0.063	0.059	0.059	0.062	0.054	0.050
	256	0.209	0.189	0.064	0.049	0.052	0.049	0.050	0.058	0.055
$LN(0,2)$	8	0.099	0.090	0.057	0.057	0.058	0.059	0.054	0.057	0.021
	32	0.166	0.158	0.057	0.061	0.058	0.057	0.053	0.057	0.056
	64	0.205	0.183	0.037	0.043	0.043	0.042	0.046	0.042	0.033
	128	0.240	0.217	0.063	0.055	0.056	0.059	0.052	0.067	0.045
	192	0.268	0.249	0.052	0.059	0.057	0.053	0.062	0.061	0.045
	256	0.294	0.264	0.053	0.051	0.048	0.041	0.042	0.050	0.062
$N(0,1)$	8	0.381	0.353	0.202	0.371	0.385	0.396	0.389	0.354	0.063
contaminated	32	0.239	0.207	0.092	0.187	0.195	0.201	0.197	0.167	0.058
$\sigma_e = 5$	64	0.155	0.145	0.070	0.114	0.107	0.111	0.101	0.107	0.074
	128	0.115	0.108	0.053	0.090	0.084	0.093	0.089	0.084	0.051
	192	0.088	0.083	0.053	0.071	0.075	0.069	0.082	0.077	0.046
	256	0.072	0.069	0.055	0.063	0.054	0.059	0.059	0.056	0.050
$N(0,1)$	8	0.629	0.605	0.394	0.620	0.624	0.642	0.634	0.592	0.069
contaminated	32	0.489	0.453	0.163	0.287	0.295	0.301	0.304	0.266	0.057
$\sigma_e = 10$	64	0.374	0.332	0.100	0.166	0.157	0.158	0.165	0.146	0.066
	128	0.256	0.228	0.069	0.121	0.111	0.118	0.115	0.100	0.049
	192	0.196	0.172	0.061	0.079	0.081	0.081	0.089	0.089	0.047
	256	0.153	0.137	0.053	0.062	0.048	0.055	0.062	0.062	0.050
