# Supplementary material for: On Robust Association Testing for Quantitative Traits and Rare Variants
Source: G3 (Bethesda). 2016 Sep 27;6(12):3941–50. doi: 10.1534/g3.116.035485 (PMC5144964; doi:10.1534/g3.116.035485)
Supplement: Supplemental Material [file supp_g3.116.035485_TableS3.pdf]

Table S3: Empirical type I error rates of various tests at the significance level of 0.05 for a quantitative trait with an error distribution (Distr), a number of correlated SNVs (#SNVs) and with two covariates.

Distr	#SNVs	SKAT	SKAT-O	SPU(1)	SPU(2)	SPU(3)	SPU(4)	SPU(∞)	aSPU	aSPUr
$N(0,1)$	8	0.052	0.051	0.053	0.056	0.049	0.049	0.048	0.053	0.056
	32	0.047	0.040	0.043	0.049	0.029	0.032	0.035	0.040	0.041
	64	0.049	0.051	0.046	0.054	0.065	0.065	0.062	0.064	0.055
	128	0.044	0.038	0.046	0.049	0.046	0.060	0.062	0.052	0.046
	192	0.023	0.037	0.057	0.031	0.053	0.043	0.061	0.045	0.049
	256	0.036	0.046	0.057	0.048	0.047	0.055	0.053	0.056	0.049
t_3	8	0.063	0.063	0.040	0.039	0.041	0.041	0.043	0.038	0.044
	32	0.103	0.097	0.043	0.046	0.044	0.036	0.051	0.039	0.053
	64	0.110	0.097	0.053	0.048	0.055	0.055	0.060	0.055	0.039
	128	0.139	0.135	0.061	0.048	0.044	0.051	0.046	0.051	0.053
	192	0.125	0.103	0.040	0.033	0.039	0.038	0.049	0.047	0.051
	256	0.139	0.119	0.050	0.019	0.032	0.025	0.041	0.035	0.052
t_1	8	0.076	0.070	0.052	0.047	0.051	0.048	0.048	0.046	0.042
	32	0.156	0.148	0.054	0.053	0.059	0.064	0.076	0.060	0.046
	64	0.205	0.198	0.051	0.043	0.046	0.045	0.079	0.061	0.026
	128	0.244	0.229	0.057	0.041	0.048	0.048	0.073	0.059	0.041
	192	0.277	0.257	0.046	0.026	0.029	0.032	0.074	0.050	0.033
	256	0.273	0.253	0.053	0.030	0.035	0.036	0.070	0.055	0.039
$LN(0,1)$	8	0.093	0.084	0.050	0.054	0.060	0.059	0.061	0.059	0.048
	32	0.113	0.093	0.036	0.032	0.043	0.046	0.048	0.048	0.052
	64	0.142	0.125	0.053	0.048	0.049	0.044	0.054	0.052	0.063
	128	0.175	0.151	0.050	0.040	0.049	0.053	0.050	0.045	0.052
	192	0.182	0.157	0.057	0.028	0.035	0.042	0.053	0.049	0.050
	256	0.184	0.152	0.057	0.027	0.040	0.037	0.047	0.051	0.058
$LN(0,2)$	8	0.089	0.084	0.056	0.060	0.062	0.066	0.065	0.062	0.048
	32	0.135	0.119	0.037	0.037	0.045	0.044	0.054	0.047	0.060
	64	0.182	0.173	0.049	0.047	0.051	0.049	0.060	0.047	0.068
	128	0.247	0.220	0.045	0.043	0.057	0.055	0.055	0.049	0.059
	192	0.275	0.247	0.045	0.027	0.038	0.042	0.060	0.053	0.061
	256	0.274	0.245	0.049	0.025	0.027	0.032	0.051	0.045	0.079
$N(0,1)$	8	0.374	0.347	0.203	0.362	0.374	0.386	0.377	0.351	0.068
contaminated	32	0.230	0.197	0.097	0.171	0.191	0.195	0.194	0.159	0.058
$\sigma_e = 5$	64	0.161	0.137	0.062	0.110	0.098	0.108	0.126	0.092	0.056
	128	0.122	0.098	0.061	0.063	0.078	0.064	0.088	0.059	0.050
	192	0.084	0.076	0.049	0.052	0.072	0.067	0.080	0.063	0.048
	256	0.081	0.083	0.053	0.051	0.063	0.049	0.056	0.050	0.048
$N(0,1)$	8	0.620	0.592	0.393	0.607	0.605	0.626	0.628	0.578	0.075
contaminated	32	0.460	0.422	0.161	0.289	0.293	0.302	0.311	0.261	0.065
$\sigma_e = 10$	64	0.375	0.316	0.092	0.160	0.161	0.167	0.189	0.143	0.055
	128	0.243	0.215	0.068	0.077	0.086	0.087	0.127	0.089	0.049
	192	0.188	0.167	0.055	0.069	0.073	0.075	0.094	0.076	0.049
	256	0.177	0.166	0.068	0.043	0.052	0.050	0.061	0.056	0.048
