# Supplementary material for: On Robust Association Testing for Quantitative Traits and Rare Variants
Source: G3 (Bethesda). 2016 Sep 27;6(12):3941–50. doi: 10.1534/g3.116.035485 (PMC5144964; doi:10.1534/g3.116.035485)
Supplement: Supplemental Material [file supp_g3.116.035485_TableS4.pdf]

Table S4: Empirical type I error rates of various tests at the significance level of 0.05 after winsorizing or trimming (at level $\alpha_1 = 0.05$ or 0.025) a quantitaive trait with an error distribution (Distr). There are NO covariates. The number of independent SNVs is indicated by #SNVs.

			Winsorizing					Trimming				
Distr	α_1	#SNVs	SKAT	SKAT-O	SPU(1)	aSPU	aSPUr	SKAT	SKAT-O	SPU(1)	aSPU	aSPUr
$N(0,1)$	0.05	8	0.048	0.051	0.053	0.055	0.058	0.046	0.056	0.068	0.061	0.052
		64	0.034	0.046	0.054	0.051	0.051	0.029	0.043	0.061	0.051	0.049
		128	0.024	0.029	0.042	0.041	0.043	0.022	0.027	0.039	0.044	0.047
		256	0.013	0.033	0.042	0.052	0.054	0.014	0.038	0.049	0.057	0.055
	0.025	8	0.051	0.049	0.053	0.059	0.058	0.053	0.050	0.047	0.058	0.056
		64	0.035	0.051	0.054	0.053	0.058	0.031	0.042	0.060	0.057	0.056
		128	0.024	0.027	0.041	0.036	0.042	0.023	0.026	0.049	0.042	0.044
		256	0.015	0.032	0.046	0.050	0.048	0.014	0.036	0.050	0.045	0.050
t_1	0.05	8	0.052	0.056	0.047	0.045	0.059	0.060	0.058	0.052	0.048	0.049
		64	0.045	0.046	0.051	0.046	0.056	0.055	0.052	0.036	0.044	0.053
		128	0.043	0.053	0.053	0.053	0.054	0.050	0.047	0.046	0.061	0.042
	0.025	8	0.080	0.070	0.045	0.048	0.059	0.089	0.069	0.051	0.051	0.054
		64	0.099	0.082	0.057	0.050	0.055	0.112	0.090	0.035	0.049	0.042
		128	0.079	0.072	0.047	0.061	0.055	0.085	0.080	0.051	0.050	0.054
$LN(0,2)$	0.05	8	0.081	0.060	0.048	0.045	0.056	0.093	0.072	0.052	0.053	0.053
		64	0.080	0.072	0.054	0.052	0.047	0.100	0.071	0.051	0.047	0.052
		128	0.071	0.064	0.060	0.056	0.047	0.106	0.080	0.052	0.044	0.052
	0.025	8	0.101	0.074	0.042	0.043	0.060	0.109	0.090	0.046	0.045	0.059
		64	0.116	0.097	0.047	0.055	0.045	0.139	0.125	0.067	0.060	0.043
		128	0.127	0.098	0.045	0.061	0.044	0.152	0.117	0.058	0.049	0.049
Contam. $\sigma_e = 10$	0.05	8	0.068	0.070	0.069	0.079	0.066	0.043	0.056	0.057	0.060	0.050
		64	0.037	0.048	0.058	0.055	0.054	0.031	0.045	0.061	0.047	0.046
		128	0.024	0.022	0.041	0.042	0.041	0.023	0.024	0.039	0.045	0.043
	0.025	8	0.085	0.082	0.072	0.095	0.074	0.049	0.051	0.051	0.061	0.056
		64	0.039	0.053	0.054	0.057	0.059	0.037	0.043	0.056	0.053	0.061
		128	0.024	0.026	0.042	0.043	0.046	0.021	0.028	0.050	0.039	0.043
