# Supplementary material for: On Robust Association Testing for Quantitative Traits and Rare Variants
Source: G3 (Bethesda). 2016 Sep 27;6(12):3941–50. doi: 10.1534/g3.116.035485 (PMC5144964; doi:10.1534/g3.116.035485)
Supplement: Supplemental Material [file supp_g3.116.035485_TableS5.pdf]

Table S5: Empirical type I error rates of various tests at the significance level of 0.05 after winsorizing or trimming (at level $\alpha_1 = 0.05$ or 0.025) a quantitaive trait with an error distribution (Distr). There are TWO covariates. The number of independent SNVs is indicated by #SNVs.

			Winsorizing					Trimming				
Distr	α_1	#SNVs	SKAT	SKAT-O	SPU(1)	aSPU	aSPUr	SKAT	SKAT-O	SPU(1)	aSPU	aSPUr
$N(0,1)$	0.05	8	0.041	0.047	0.046	0.054	0.028	0.040	0.049	0.057	0.054	0.038
		64	0.051	0.049	0.047	0.049	0.019	0.042	0.039	0.047	0.052	0.030
		128	0.035	0.045	0.054	0.048	0.015	0.034	0.037	0.055	0.045	0.029
	0.025	8	0.043	0.049	0.051	0.053	0.039	0.037	0.040	0.047	0.051	0.036
		64	0.042	0.039	0.050	0.048	0.034	0.042	0.035	0.044	0.050	0.040
		128	0.042	0.038	0.053	0.048	0.036	0.028	0.037	0.051	0.048	0.038
t_1	0.05	8	0.080	0.076	0.063	0.060	0.043	0.082	0.078	0.051	0.061	0.047
		64	0.077	0.068	0.052	0.061	0.035	0.084	0.069	0.043	0.043	0.030
		128	0.067	0.067	0.060	0.049	0.042	0.066	0.067	0.054	0.047	0.032
	0.025	8	0.089	0.083	0.060	0.049	0.052	0.106	0.091	0.056	0.061	0.050
		64	0.091	0.080	0.042	0.042	0.039	0.114	0.099	0.053	0.058	0.041
		128	0.091	0.092	0.061	0.052	0.052	0.103	0.089	0.055	0.045	0.050
$LN(0,2)$	0.05	8	0.080	0.073	0.059	0.053	0.044	0.116	0.092	0.048	0.052	0.040
		64	0.076	0.066	0.057	0.046	0.043	0.105	0.086	0.058	0.056	0.042
		128	0.073	0.055	0.039	0.041	0.045	0.093	0.071	0.037	0.033	0.030
	0.025	8	0.096	0.076	0.053	0.051	0.051	0.130	0.111	0.052	0.061	0.049
		64	0.109	0.090	0.051	0.045	0.062	0.129	0.103	0.038	0.041	0.044
		128	0.101	0.069	0.049	0.040	0.066	0.148	0.122	0.049	0.044	0.051
Contam.	0.05	8	0.327	0.294	0.155	0.282	0.104	0.115	0.111	0.081	0.105	0.061
$\sigma_e = 10$		64	0.118	0.083	0.054	0.069	0.027	0.054	0.051	0.044	0.056	0.033
		128	0.070	0.070	0.056	0.055	0.015	0.039	0.045	0.057	0.045	0.026
	0.025	8	0.405	0.377	0.186	0.333	0.141	0.137	0.127	0.082	0.125	0.067
		64	0.144	0.103	0.054	0.077	0.045	0.064	0.051	0.045	0.057	0.043
		128	0.092	0.071	0.056	0.052	0.034	0.043	0.043	0.054	0.055	0.038
