# Supplementary material for: On Robust Association Testing for Quantitative Traits and Rare Variants
Source: G3 (Bethesda). 2016 Sep 27;6(12):3941–50. doi: 10.1534/g3.116.035485 (PMC5144964; doi:10.1534/g3.116.035485)
Supplement: Supplemental Material [file supp_g3.116.035485_TableS6.pdf]

Table S6: Empirical power of various tests at the significance level of 0.05 for a quantitative trait and a number of independent SNVs (#SNVs), without any covariates. Cases I-V correspond to causal SNVs with non-zero $\beta = (-1.2, -1.2, -0.8, -0.8, 0.8, 1, 1, 1)'$, $\beta = (0.7, 0.7, 0.7, 1, 1, 1, 1.2, 1.2)'$, $\beta = (3, runif(7, 0.3, 0.5))'$, $\beta = (2, 2, runif(6, 0.1, 0.3))'$, $\beta = (2, 2, 2, runif(5, 0, 0.2))'$, respectively.

Case	#SNVs	SKAT	SKAT-O	SPU(1)	SPU(2)	SPU(3)	SPU(4)	SPU(∞)	aSPU	aSPUr
I	8	0.858	0.809	0.186	0.854	0.596	0.788	0.696	0.808	0.734
	32	0.652	0.564	0.079	0.655	0.370	0.571	0.465	0.553	0.439
	64	0.466	0.379	0.084	0.468	0.297	0.454	0.380	0.420	0.322
	128	0.316	0.245	0.058	0.364	0.206	0.366	0.287	0.316	0.231
	192	0.225	0.143	0.046	0.299	0.166	0.307	0.241	0.259	0.175
	256	0.173	0.128	0.052	0.258	0.173	0.297	0.241	0.250	0.170
II	8	0.846	0.953	0.968	0.836	0.856	0.772	0.689	0.945	0.926
	32	0.608	0.649	0.512	0.609	0.602	0.537	0.450	0.622	0.527
	64	0.448	0.455	0.302	0.470	0.450	0.432	0.344	0.466	0.373
	128	0.305	0.315	0.184	0.352	0.344	0.349	0.279	0.368	0.286
	192	0.223	0.195	0.123	0.286	0.282	0.311	0.251	0.299	0.203
	256	0.167	0.153	0.109	0.246	0.238	0.279	0.226	0.279	0.185
III	8	0.734	0.777	0.739	0.707	0.723	0.691	0.672	0.763	0.556
	32	0.579	0.567	0.326	0.562	0.597	0.592	0.579	0.587	0.327
	64	0.460	0.449	0.189	0.449	0.496	0.515	0.508	0.506	0.227
	128	0.356	0.325	0.128	0.371	0.426	0.444	0.436	0.428	0.196
	192	0.277	0.251	0.094	0.308	0.405	0.434	0.405	0.408	0.151
	256	0.253	0.208	0.080	0.302	0.360	0.415	0.412	0.390	0.154
IV	8	0.766	0.791	0.714	0.748	0.760	0.733	0.715	0.778	0.613
	32	0.604	0.559	0.298	0.576	0.618	0.613	0.585	0.603	0.389
	64	0.459	0.424	0.167	0.457	0.479	0.490	0.479	0.481	0.282
	128	0.340	0.291	0.115	0.367	0.415	0.437	0.417	0.440	0.250
	192	0.247	0.203	0.083	0.307	0.361	0.413	0.378	0.379	0.182
	256	0.205	0.182	0.073	0.288	0.322	0.398	0.373	0.360	0.190
V	8	0.885	0.905	0.839	0.874	0.878	0.858	0.834	0.880	0.746
	32	0.751	0.731	0.421	0.727	0.728	0.736	0.680	0.733	0.536
	64	0.619	0.561	0.242	0.607	0.618	0.647	0.604	0.624	0.406
	128	0.487	0.444	0.153	0.523	0.568	0.588	0.560	0.570	0.320
	192	0.382	0.322	0.118	0.429	0.500	0.540	0.507	0.513	0.255
	256	0.316	0.253	0.090	0.395	0.432	0.501	0.488	0.489	0.255
