# Supplementary material for: On Robust Association Testing for Quantitative Traits and Rare Variants
Source: G3 (Bethesda). 2016 Sep 27;6(12):3941–50. doi: 10.1534/g3.116.035485 (PMC5144964; doi:10.1534/g3.116.035485)
Supplement: Supplemental Material [file supp_g3.116.035485_TableS7.pdf]

Table S7: Empirical power of various tests at the significance level of 0.05 for a quantitaive trait and a number of correlated SNVs (#SNVs), with two covariates. Cases I-II correspond to causal SNVs with non-zero $\beta = (-1.2,\ -1.2,\ -0.8,\ -0.8,\ 0.8,\ 1,\ 1,\ 1)'$, $\beta = (0.7,\ 0.7,\ 0.7,\ 1,\ 1,\ 1,\ 1.2,\ 1.2)'$, respectively.

Case	#SNVs	SKAT	SKAT-O	SPU(1)	SPU(2)	SPU(3)	SPU(4)	SPU(∞)	aSPU	aSPUr
I	8	0.915	0.897	0.391	0.917	0.771	0.894	0.863	0.887	0.790
	32	0.752	0.685	0.168	0.741	0.473	0.698	0.596	0.671	0.550
	64	0.603	0.522	0.124	0.602	0.373	0.562	0.478	0.526	0.400
	128	0.413	0.333	0.085	0.425	0.274	0.421	0.337	0.373	0.275
	192	0.334	0.249	0.079	0.354	0.217	0.383	0.287	0.324	0.235
	256	0.228	0.187	0.069	0.245	0.165	0.297	0.254	0.253	0.194
II	8	0.968	0.983	0.988	0.968	0.964	0.947	0.921	0.980	0.957
	32	0.793	0.834	0.793	0.786	0.797	0.738	0.634	0.821	0.712
	64	0.627	0.648	0.551	0.624	0.665	0.602	0.493	0.652	0.542
	128	0.407	0.434	0.345	0.418	0.470	0.418	0.328	0.451	0.347
	192	0.321	0.335	0.271	0.331	0.385	0.361	0.278	0.374	0.298
	256	0.238	0.257	0.215	0.251	0.322	0.301	0.242	0.320	0.240
