# Supplementary material for: On Robust Association Testing for Quantitative Traits and Rare Variants
Source: G3 (Bethesda). 2016 Sep 27;6(12):3941–50. doi: 10.1534/g3.116.035485 (PMC5144964; doi:10.1534/g3.116.035485)
Supplement: Supplemental Material [file supp_g3.116.035485_TableS8.pdf]

Table S8: Empirical power of various tests at the significance level of 0.05 after winsorizing or trimming (at level $\alpha_1 = 0.05$ or 0.025) a quantitaive trait. There are NO covariates; Cases I-II correspond to causal SNVs with non-zero $\beta = (-1.2, -1.2, -0.8, -0.8, 0.8, 1, 1, 1)'$, $\beta = (0.7, 0.7, 0.7, 1, 1, 1, 1.2, 1.2)'$, respectively.

			Winsorizing					Trimming				
Distr	α_1	#SNVs	SKAT	SKAT-O	SPU(1)	aSPU	aSPUr	SKAT	SKAT-O	SPU(1)	aSPU	aSPUr
I	0.05	8	0.836	0.759	0.154	0.770	0.749	0.468	0.377	0.112	0.416	0.400
		64	0.360	0.263	0.061	0.324	0.300	0.143	0.101	0.049	0.150	0.143
		128	0.211	0.152	0.056	0.239	0.224	0.081	0.072	0.059	0.106	0.108
	0.025	8	0.863	0.792	0.165	0.785	0.741	0.640	0.535	0.126	0.550	0.546
		64	0.400	0.286	0.067	0.357	0.290	0.200	0.138	0.059	0.189	0.169
		128	0.240	0.173	0.060	0.266	0.219	0.118	0.099	0.058	0.137	0.121
II	0.05	8	0.825	0.944	0.955	0.940	0.931	0.448	0.689	0.746	0.677	0.671
		64	0.345	0.359	0.273	0.396	0.365	0.130	0.162	0.144	0.182	0.176
		128	0.204	0.212	0.174	0.305	0.265	0.069	0.098	0.106	0.113	0.114
	0.025	8	0.852	0.951	0.960	0.945	0.933	0.598	0.826	0.873	0.804	0.802
		64	0.377	0.398	0.285	0.426	0.377	0.211	0.217	0.177	0.247	0.219
		128	0.227	0.227	0.180	0.315	0.267	0.102	0.134	0.137	0.166	0.150
