# Supplementary material for: On Robust Association Testing for Quantitative Traits and Rare Variants
Source: G3 (Bethesda). 2016 Sep 27;6(12):3941–50. doi: 10.1534/g3.116.035485 (PMC5144964; doi:10.1534/g3.116.035485)
Supplement: Supplemental Material [file supp_g3.116.035485_TableS9.pdf]

Table S9: Empirical power of various tests at the significance level of 0.05 after winsorizing or trimming (at level $\alpha_1 = 0.05$ or 0.025) a quantitaive trait. There are TWO covariates; Cases I-II correspond to causal SNVs with non-zero $\beta = (-1.2, -1.2, -0.8, -0.8, 0.8, 1, 1, 1)'$, $\beta = (0.7, 0.7, 0.7, 1, 1, 1, 1.2, 1.2)'$, respectively.

			Winsorizing					Trimming				
Distr	α_1	#SNVs	SKAT	SKAT-O	SPU(1)	aSPU	aSPUr	SKAT	SKAT-O	SPU(1)	aSPU	aSPUr
I	0.05	8	0.800	0.754	0.161	0.712	0.588	0.804	0.753	0.182	0.743	0.648
		64	0.377	0.303	0.075	0.328	0.164	0.401	0.328	0.080	0.345	0.229
		128	0.239	0.169	0.052	0.233	0.103	0.242	0.166	0.041	0.233	0.135
	0.025	8	0.839	0.790	0.192	0.778	0.716	0.843	0.791	0.186	0.778	0.732
		64	0.422	0.347	0.074	0.365	0.261	0.418	0.361	0.092	0.378	0.289
		128	0.272	0.194	0.050	0.259	0.179	0.264	0.182	0.041	0.249	0.167
II	0.05	8	0.772	0.896	0.906	0.882	0.834	0.779	0.901	0.921	0.894	0.864
		64	0.339	0.361	0.260	0.353	0.208	0.352	0.363	0.246	0.352	0.248
		128	0.216	0.207	0.145	0.265	0.138	0.206	0.202	0.146	0.260	0.161
	0.025	8	0.815	0.922	0.934	0.917	0.890	0.823	0.933	0.944	0.917	0.900
		64	0.396	0.404	0.270	0.404	0.318	0.393	0.402	0.260	0.411	0.340
		128	0.240	0.244	0.159	0.304	0.214	0.242	0.222	0.160	0.302	0.216
